# Relationship between the triglyceride-glucose index and risk of cardiovascular diseases and mortality in the general population: a systematic review and meta-analysis

**DOI:** 10.1186/s12933-022-01546-0

**Published:** 2022-07-01

**Authors:** Xiao Liu, Ziqi Tan, Yuna Huang, Huilei Zhao, Menglu Liu, Peng Yu, Jianyong Ma, Yujie Zhao, Wengen Zhu, Jingfeng Wang

**Affiliations:** 1 Department of Cardiology, Sun Yat-Sen Memorial Hospital of Sun Yat-Sen University, Guangzhou, 510080 Guangdong China; 2 Guangdong Province Key Laboratory of Arrhythmia and Electrophysiology, Guangzhou, 510120 Guangdong China; 3 Department of Endocrine, The Second Affiliated Hospital of Nanchang University, Nanchang, 330006 Jiangxi China; 4 Department of Anesthesiology, The Third Hospital of Nanchang, Nanchang, 330006 Jiangxi China; 5 Department of Cardiology, Seventh People’s Hospital of Zhengzhou, Zhengzhou, 334000 Henan China; 6 Department of Pharmacology and Systems Physiology, University of Cincinnati College of Medicine, Cincinnati, OH 45267 USA; 7 Department of Cardiology, The First Affiliated Hospital of Sun Yat-Sen University, Guangzhou, 510080 Guangdong China

**Keywords:** Triglyceride-glucose index, Cardiovascular disease, Coronary artery disease, Mortality, Meta-analysis

## Abstract

**Background:**

The triglyceride-glucose (TyG) index is a new alternative measure for insulin resistance. This meta-analysis was conducted to assess the associations of the TyG index with the risks of cardiovascular diseases and mortality in the general population.

**Methods:**

The PubMed, Cochrane Library and Embase databases were searched for randomized controlled trials or observational cohort studies reporting associations of the TyG index with cardiovascular diseases and mortality from inception to April 16, 2022. Effect sizes were pooled using random-effects models. Robust error meta-regression methods were applied to fit nonlinear dose–response associations. Evidence quality levels and recommendations were assessed using the Grading of Recommendations Assessment, Development and Evaluation system (GRADE).

**Results:**

Twelve cohort studies (6 prospective and 6 retrospective cohorts) involving 6,354,990 participants were included in this meta-analysis. Compared with the lowest TyG index category, the highest TyG index was related to a higher incidence of coronary artery disease (CAD) (3 studies; hazard ratio [HR] = 2.01; 95% confidence interval [CI] 1.68–2.40; I^2^ = 0%), myocardial infarction (MI) (2 studies; HR = 1.36; 95% CI 1.18–1.56; I^2^ = 35%), and composite cardiovascular disease (CVD) (5 studies; HR = 1.46; 95% CI 1.23–1.74; I^2^ = 82%). However, there was no association between the TyG index and mortality (cardiovascular mortality [3 studies; HR = 1.10; 95% CI 0.82–1.47; I^2^ = 76%] or all-cause mortality [4 studies; HR = 1.08; 95% CI 0.92–1.27; I^2^ = 87%]). In the dose–response analysis, there was a linear association of the TyG index with the risk of CAD (P_nonlinear_ = 0.3807) or CVD (P_nonlinear_ = 0.0612). GRADE assessment indicated very low certainty for CVD, MI, cardiovascular mortality and all-cause mortality, and moderate certainty for CAD.

**Conclusions:**

Based on our current evidence, a higher TyG index may be associated with an increased incidence of CAD (moderate certainty), MI (very low certainty) and CVD (very low certainty) in the general population. There is a potential linear association of the TyG index with CAD and the composite CVD incidence. Further prospective studies (especially in non-Asians) are needed to confirm our findings.

**Supplementary Information:**

The online version contains supplementary material available at 10.1186/s12933-022-01546-0.

## Introduction

Cardiovascular disease (CVD) is the leading cause of premature death worldwide and a major contributor to the global burden of disease [1]. According to the World Health Organization (WHO), CVD accounts for the majority of deaths from noncommunicable diseases [2]. Diabetes, hypertension, obesity, and smoking are recognized as important risk factors for cardiovascular disease [3, 4]. Insulin resistance is the main feature and has a significant pathogenic link to type 2 diabetes mellitus (T2DM) [5, 6]. For example, a recent study showed that people with insulin-resistant diabetes have a high risk of developing cardiovascular disease [2]. The triglyceride-glucose (TyG) index is a synthetic parameter of fasting glucose in healthy individuals and is considered a reliable surrogate marker of insulin resistance [7, 8]. As a novel method, the TyG index is expected to become an alternative index for the hyperinsulinemic-euglycemic (HIEG) clamp test, a traditional method for assessing insulin sensitivity [9, 10].

As an indicator of insulin resistance, a high TyG index has been shown to be associated with adverse cardiovascular events among patients with diabetes and CVD, such as stable coronary artery disease (CAD) [11], acute coronary syndrome [8, 12] and after percutaneous coronary intervention [13, 14]. In addition, recent studies report a predictive role for the TyG index with regard to atherosclerosis [15], myocardial infarction (MI) [16] and CAD [17] in patients without diabetes and in the general population. A community-based cohort study of 7521 participants in Iran determined that a high TyG index was significantly associated with an increased risk of CVD/CAD after 3 years of follow-up [18], and the results of the Chinese Kailuan study demonstrated a correlation between the TyG index and MI [19–21]. These findings suggest that the TyG index might serve as a cardiovascular event marker independent of traditional risk factors in the general population. Accordingly, the aim of this study was to summarize the relationship and potential dose–response associations of the TyG index with CVD and mortality risk in the general population.

## Methods

The protocol was registered with PROSPERO (International Prospective Register of Systematic Reviews. http: www.york.ac.uk/inst/crd) under registration number CRD42022296235. This meta-analysis was conducted following Preferred Reporting Item for Systematic Review and Meta-Analysis 2020 guidelines (PRISMA 2020) (Additional file 1: Table S1).

### Literature search

The PubMed, Embase, and the Cochrane Library databases were searched for studies from inception to April 16, 2022, without language restrictions. The search terms were as follows: (1) Exposure: For the TyG index: “TyG index” or “triglyceride-glucose index”. (2) Outcomes: For CVD: “cardiovascular disease”, “cardiovascular diseases”, “coronary artery disease”, “coronary disease”, “coronary arteriosclerosis”, “myocardial infarction”, “heart attack”, “heart failure”, “heart decompensation”, “atrial fibrillation”, “sudden cardiac death”, “arrhythmia”, “cardiomyopathy”, “hypertrophic cardiomyopathy”, and “dilated cardiomyopathy”. For mortality: “mortality”, “death”, “cardiovascular death”, and “all-cause mortality”. We did not apply keywords for the population and study design in the database search to capture as many studies as possible that met the inclusion criteria. The detailed search strategy is described in Additional file 1: Table S2.

### Study selection

We used Endnote X9 software (Thomson Reuters, New York, NY, USA) to organize all studies. After removing duplicates, titles and abstracts were checked, and an initial screening of the relevant literature was performed, and the full text of studies was retrieved for full-text assessment of eligibility for inclusion.

According to the PICOS (population, intervention, comparison, outcome, and study design), the criteria for considering studies for this review were as follows: (1) types of participants: adult (age > 18 years) general population. (2) Exposure and comparator: high versus low TyG index level. (3) Outcomes: all kinds of cardiovascular diseases, composite of cardiovascular disease, cardiovascular mortality, and all-cause mortality. (4) Types of studies: random controlled trails (RCTs), post hoc analyses of RCTs, or observational cohort studies.

Our exclusion criteria included: Cross-sectional studies due to the high risk for bias; Studies reporting the association of the TyG index with prognosis in established conditions (e.g., cardiovascular diseases, diabetes); Studies focusing on children and adolescents; Studies not reporting multivariable adjusted outcomes. In addition, articles without sufficient data (reviews, editorials, preclinical studies) and studies not relevant for the purpose of the current meta-analysis were excluded. If the same population was used in multiple studies, we selected the article with the most information or the largest sample size.

### Data extraction and quality assessment

Literature searches, data extraction and quality assessment of the included studies were conducted independently by two authors (Z. Q-T and X-L) based on predetermined criteria. The following data were extracted: (1) author names, year of publication, and country; (2) study design and duration of follow-up; (3) study population characteristics, including data source, sample size, mean age, and sex; (4) outcomes and determination of outcomes; and (5) risk estimates and adjustments.

The Newcastle–Ottawa Scale (NOS) was used to assess the quality of included cohort studies. Scores ranged from 0 to 9 to assess article selection, comparability, and outcomes. Studies with an NOS score of > 6 were considered to be of high quality [22].

### Statistical analysis

We used RevMan software version 5.4 (The Cochrane Collaboration 2014, Nordic Cochrane Center Copenhagen, Denmark) for statistics and analysis. We used risk estimates of hazard ratios (HRs) and their corresponding 95% confidence intervals (CIs) as general indicators of the association between the TyG index and cardiovascular disease in the general population. The TyG index was calculated using the following equation: TyG = Ln (TG [mg/dL] × fasting glucose [mg/dL]/2) [23, 24]. The TyG index was analyzed as a categorical variable with the highest TyG index levels compared to the group with the lowest TyG index levels. We also conducted a supplemental analysis of the moderate (if there were four groups, we chose the third highest TyG index level group) versus lowest TyG index levels. If the article reported sex subgroups, we first used a random effects model to combine to obtain effect sizes. The TyG index was analyzed as a continuous variable, and the units of the TyG index (per 1 unit) were standardized. We estimated linear trends and 95% CIs from the natural logarithm of the effect size and CI for the TyG index category, as described by Greenland and Longnecker [25]. A random-effects model was used to improve reliability [26]. Robust error meta-regression methods were used to fit nonlinear dose–response analysis described by Xu and Doi et al*.* [27, 28]. This method is based on a “one-stage approach”, which treats each study as a cluster of the whole sample and considers the within-study correlations by clustered robust error. It requires known levels of the TyG index and risk estimates with variance estimates for at least two quantitative exposure categories [12]. As described in previous articles [29, 30], if quantitative TyG values were not reported, we used the median or mean TyG of the categories. If the median or mean TyG was not provided and reported in ranges, we estimated the midpoint of each category by averaging the lower and upper boundaries of that category. If the highest or lowest category was open-ended, we assumed that the open-ended interval length was the same as the adjacent interval [14].

We assessed the degree of heterogeneity using the I^2^ test, whereby 25%, 50%, and 75% represented low, moderate, and high heterogeneity, respectively [29]. Publication bias was evaluated using funnel plots. Sensitivity analyses were performed by omitting each study in turn. Subgroup analyses were performed stratified by sex, age, and diabetes if the number of included studies exceeded ten for each outcome [31]. P < 0.05 was considered statistically significant.

### Quality of evidence

We assessed the quality and strength of the evidence for each outcome according to the Grading of Recommendations Assessment, Development and Evaluation (GRADE) method [32, 33]. Two authors rated the quality of the evidence for each outcome separately. We used GRADEprofiler software and provide evidence profile tables. We present the results of the outcomes as described in the outcome metric type section; footnotes are used to justify all decisions to lower or raise the quality of evidence.

## Results

### Literature search

**Fig. 1:**
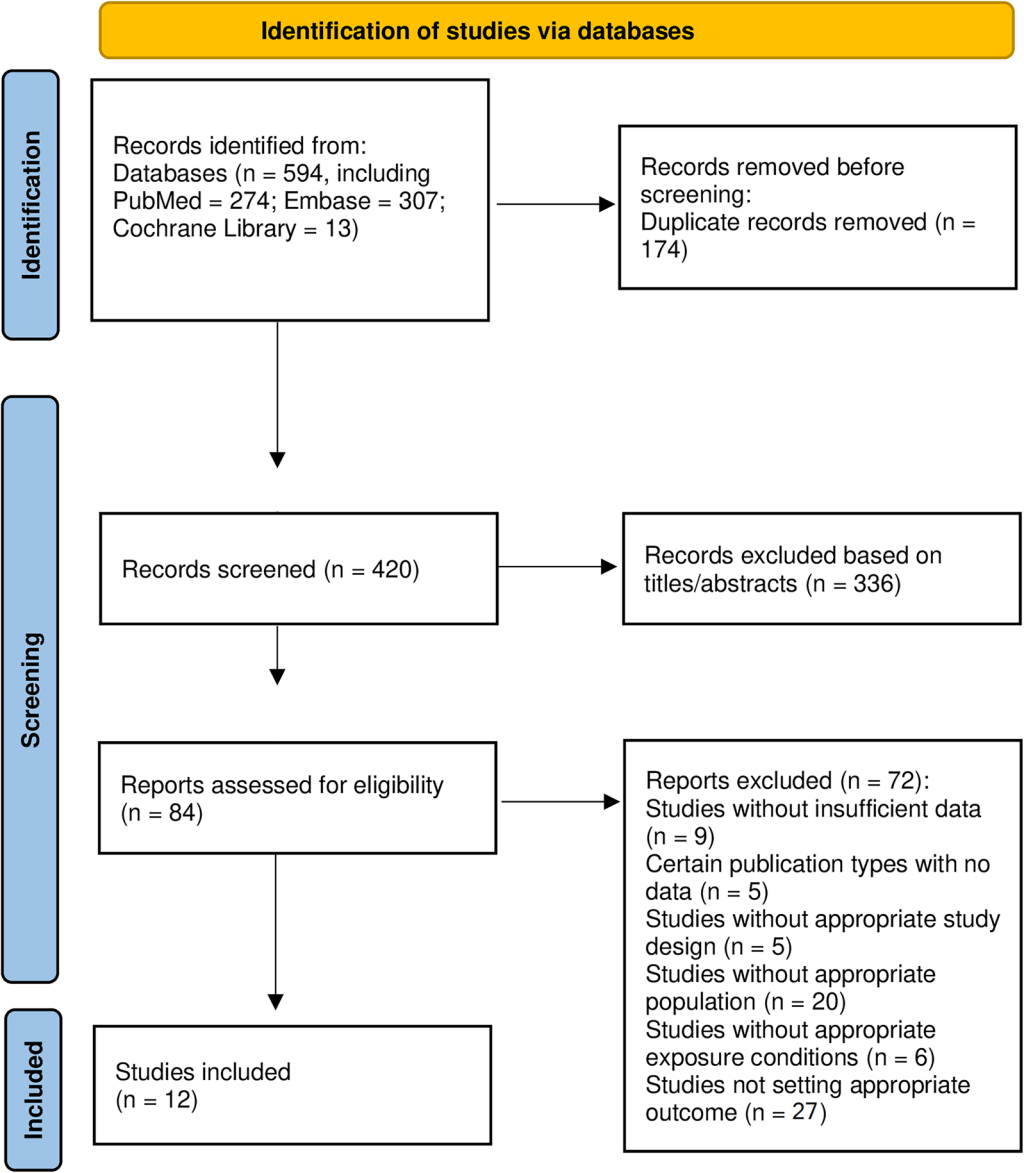
Flowchart of the study selection for the meta-analysis for the association between TyG index and cardiovascular diseases and mortality in general population.\ Abbreviation: TyG: Triglyceride-glucose

The flow chart shows the process of the database search (Fig. 1). An initial search of 594 articles (PubMed = 274; Embase = 307; Cochrane Library = 13) was conducted according to the search protocol. Of these, 174 duplicate articles were excluded; in addition, 336 articles were excluded as irrelevant after screening titles and abstracts. Subsequently, the full texts of 84 articles were reviewed. Of these, 72 were further excluded for the following reasons: (1) insufficient data (n = 9); (2) certain publication type for which no data were available (n = 5); (3) without an appropriate study design (n = 5); (4) without an appropriate population (n = 20); (5) without an appropriate exposure (n = 6); and (6) did not report the target outcome (n = 27). Ultimately, no RCT studies met the inclusion criteria, 12 cohort studies were used for this meta-analysis [18–20, 34–42]. All excluded studies and the reasons (n = 72) are shown in Additional file 1: Table S3.

### Study characteristics and quality evaluation

The basic characteristics of all included observational articles are summarized in Table 1. The twelve cohort studies [18–20, 34–42], including 6,354,990 participants with a mean age between 44.9 and 70.5 years, were published from 2014 to 2021; sample sizes ranged from 5014 to 5,593,134. Of these, three studies reported CAD, two reported MI, six reported CVD, and four reported mortality outcomes (cardiovascular [CV] mortality had three and all-cause mortality had four). The definitions of CVD in each study are listed in Additional file 1: Table S4. All articles reported the follow-up time, with eleven exceeding 5 years [18–20, 34–39, 41, 42]. The span of follow-up ranged from 4.1 years to 16.1 years. Of these studies, ten studies were performed in Asia (four in China [19, 20, 37, 38], four in Korea [34–36, 40] and two in Iran [18, 39]), one was from the Americas (The United States of America [USA] [42]) and one was from Europe (Spain [41]). In addition, six were prospective cohort studies [18–20, 39–41]; the others were retrospective cohort studies [34–38, 42].

**Table 1:** Basic characteristics of the articles included in the meta-analysis of TyG and risk of CVD and mortality in general population

Author, years, country	Study design/Mean follow-up time	Study population	Sample sizes	Mean age (years)/Male (%)	TyG detection	Endpoint detection	Endpoint	TyG index	Hazard risk (95% CI)	Adjustments
Barzegar, 2020, Iran	Prospective cohort study/16.1 years	Tehran Lipid and Glucose Study Free of CVD at baseline	7521	46.60/44.80	NA	ECG and ICD-10	CAD (incidence)	< 8.4	1	Age, gender, WC, BMI, education, smoking status, physical activity, FHCVD, T2D, hypertension, lipid lowering drugs, LDL-C, HDL-C
								8.4–8.7	1.25 (0.93–1.67)	
								8.7–9.0	1.49 (1.12–1.98)	
								9.0–9.4	1.34 (1.01–1.80)	
								≥ 9.4	1.84 (1.37–2.48)	
								Per SD (0.48)	1.19 (1.10–1.29)	
						ECG and ICD-10	CVD (incidence)	< 8.4	1	
								8.4–8.7	1.15 (0.89–1.49)	
								8.7–9.0	1.28 (0.99–1.65)	
								9.0–9.4	1.22 (0.94–1.58)	
								≥ 9.4	1.61 (1.23–2.11)	
								Per SD (0.48)	1.16 (1.07–1.25)	
Hong, 2020, Korea	Retrospective cohort study/8.2 years	National Health Information Database Free of ASCVD history, diabetes and hyperlipidemia	5,593,134	53.02/50.50	NA	ICD-10 code I21 or I22	MI (incidence)	Q1	1	Age, sex, smoking, alcohol consumption, regular physical activity, low socioeconomic status, BMI, hypertension, total cholesterol level, hypertension medications, warfarin, and aspirin
								Q2	1.09 (1.07–1.12)	
								Q3	1.17 (1.14–1.19)	
								Q4	1.31 (1.28–1.35)	
Kim, 2019, Korea	Retrospective cohort study/5.66 years	Kangbuk Samsung Health Study General population	318,224	NA/51.89	NA	Derived from the Korea National Statistical Office	CV mortality	Highest vs. lowest	1.26 (1.02–1.55)	Age, sex, BMI
						Derived from the Korea National Statistical Office	All-cause mortality	Highest vs. lowest	1.12 (1.03–1.22)	
Kim, 2021, Korea	Retrospective cohort study/5.97 years	Korean National Health Insurance Service—National Health Screening cohort Free of CVD, diabetes and tumor	144,603	56.00/53.96	NA	ICD-10 codes I20-I25	CVD (men, incidence)	< 8.25	1	Age, smoking status, drinking status, physical activity, BMI, SBP, LDL-C, economic status, and anti-hypertensive medications
								8.25–8.61	1.11 (0.97–1.28)	
								8.61–9.00	1.19 (1.03–1.36)	
								≥ 9.00	1.23 (1.07–1.42)	
							CVD (women, incidence)	< 8.06	1	
								8.06–8.40	1.24 (1.02–1.51)	
								8.40–8.75	1.18 (0.97–1.44)	
								≥ 8.75	1.24 (1.02–1.51)	
							All-cause mortality (men)	< 8.25	1	
								8.25–8.61	1.05 (0.91–1.21)	
								8.61–9.00	1.11 (0.96–1.28)	
								≥ 9.00	1.06 (0.91–1.25)	
							All-cause mortality (women)	< 8.06	1	
								8.06–8.40	1.22 (0.94–1.58)	
								8.40–8.75	1.01 (0.78–1.31)	
								≥ 8.75	0.99 (0.76–1.28)	
Li, 2019, China	Retrospective cohort study/5.52 years	Health check-up programme in Xinzheng and Xinmi City Free of history of CVD, type 1 diabetes and obesity (BMI > 45 kg/m2)	6,078	70.45/53.08	Automatic biochemical analyser	ICD-10 codes I20-I25	CAD (incidence)	< 8.32	1	Age and sex, living alone, current smoking, alcohol consumption, exercise, BMI, resting heart rate, SBP, HDL-C, LDL-C, and diabetic status, time-varying repeated measures of TyG
								8.32–8.61	1.22 (0.93–1.60)	
								8.61–8.89	1.26 (0.96–1.66)	
								≥ 8.90	2.05 (1.58–2.64)	
								Per 1 unit	1.63 (1.39–1.90)	
						Hospital dataset for admissions	CVD (incidence)	< 8.32	1	
								8.32–8.61	1.00 (0.80–1.25)	
								8.61–8.89	1.17 (0.94–1.45)	
								≥ 8.90	1.61 (1.31–1.99)	
								Per 1 unit	1.43 (1.24–1.63)	
Liu, 2020, China	Retrospective cohort study/98.20 months	National Health and Nutrition Examination Survey General population	19,420	47.10/48.90	Enzymatic assays and hexokinase method	ICD-10 codes I00–I09, I11, I13, I20–I51, I60–I69	CV mortality	≤ 8.00	1	Age, sex, race, smoking, BMI, SBP, Egfr, TC, HDL-C, comorbidities (cardiovascular disease, diabetes, and hypertension), and medicine use (hypotensive drugs, hypoglycemic drugs, lipid-lowering medication, and antiplatelet drugs)
								8.00–9.00	0.63 (0.43–0.93)	
								9.00–10.00	0.64 (0.41–1.00)	
								> 10.00	1.37 (0.78–2.42)	
								Per 1 unit	1.29 (1.05–1.57)	
						The National Center for Health Statistics with personal data and death certificate	All-cause mortality	≤ 8.00	1	
								8.00–9.00	0.93 (0.77–1.11)	
								9.00–10.00	0.88 (0.72–1.09)	
								> 10.00	1.51 (1.15–1.98)	
								Per 1 unit	1.10 (1.00–1.20)	
Liu, 2021, China	Prospective cohort study/10.33 years	Kailuan General Hospital Free of CVD and cancer history, and obesity (BMI > 45 kg/m2)	96,541	51.19/79.61	Hitachi 747 autoanalyzer	Basis of clinical symptoms and dynamic changes in cardiac enzymes and/or biomarker concentrations and electrocardiogram results	CVD (incidence)	≤ 8.18	1	Age, sex, education, and current smoking status, current drinking status, physical activity, BMI, hypertension, diabetes, HDL-C, LDL-C, hs-CRP, lipid-lowering medication, antidiabetic medication, and antihypertensive medication
								8.18–8.57	1.09 (1.02–1.18)	
								8.57–9.05	1.18 (1.09–1.27)	
								> 9.05	1.20 (1.11–1.30)	
								Per 1 unit	1.09 (1.05–1.13)	
Mirshafiei, 2021, Iran	Prospective cohort study/6.00 years	Mashhad stroke and heart association disorder study Free of diabetes, hyperlipidemia, hypertensive and CVD history	9704	48.09/40.13	NA	Clinic for confirmation and questionnaire	CVD (incidence)	Per 1 SD (0.82)	2.31 (1.93–2.76)	Sex, age, smoking, BMI, family history of CVD, HTN, diabetes, and LDL
							Cardiac mortality	Per 1 SD (0.82)	2.30 (1.25–4.24)	
Park, 2020, Korea	Prospective cohort study/50 months	A health risk assessment study Free of IHD or ischemic stroke history, type 2 diabetes or a fasting plasma glucose level ≥ 126 mg/dL, current use of dyslipidaemia medication or aspirin, and hs-CRP levels ≥ 10 mg/L	16,455	46.1/51.21	Enzymatic methods using a Hitachi 7600 Automated Chemistry Analyzer	ICD-10 codes I20 and I21	Ischemic heart disease (incidence)	≤ 8.08	1	Age, sex, body mass index, smoking status, alcohol intake, physical activity, hs-CRP level, mean arterial blood pressure, chronic kidney disease, and hypertension medication
								8.09–8.45	1.61 (1.05–2.48)	
								8.46–8.85	1.85 (1.21–2.83)	
								≥ 8.86	2.28 (1.48–3.51)	
Sanchez-Inigo, 2016, Spain	Prospective cohort study/8.75 years	Vascular Metabolic CUN cohort Free of CVD at baseline, history of type 1 diabetes or latent autoimmune diabetes in adults, cancer in the palliative phase, familial hypertriglyceridaemia, extreme BMI (> 45 kg/m2) or a hypercoagulable state	5014	54.41/61.19	Hitachi 711 Chemistry Analyzer and hexokinase method	ICD-10 code I20-I25, I63-I66, I73 and I74	CVD (incidence)	6.40–7.87	1	Age, sex, BMI, cigarette smoking, daily alcohol intake, lifestyle pattern (physically active/sedentary behavior), hypertension, T2D, anti-aggregation therapy, HDL-C, LDL-C
								7.88–8.18	1.19 (0.83–1.71)	
								8.19–8.47	1.32 (0.93–1.88)	
								8.48–8.80	1.52 (1.07–2.16)	
								8.81–12.42	2.32 (1.65–3.26)	
Tian, 2021, China	Prospective cohort study/11.03 years	Kailuan study Free of MI history	98,849	51.81/79.75	Hexokinase/glucose-6-phosphate dehydrogenase method and enzymatic colorimetric method	Combinations of chest pain symptoms, electrocardiographic signs, and cardiac enzyme levels	MI (incidence)	7.70–8.06	1	Age, sex, level of education, income, smoking, alcohol abuse, physical activity, BMI, SBP, DBP, a history of hypertension, diabetes mellitus, and dyslipidemia, antidiabetic drugs, lipid-lowering drugs, antihypertensive drugs, HDL-C, LDL-C, and hs-CRP at baseline
								8.29–8.48	1.07 (0.90–1.29)	
								8.68–8.91	1.30 (1.03–1.62)	
								9.23–9.82	1.58 (1.18–2.12)	
								Per 1 unit	1.49 (1.26–1.76)	
Vega, 2014, USA	Retrospective cohort study/14.75 years	Cooper Center Longitudinal Study General population	39,447	44.90/100.00	NA	ICD-9 codes 390Y449.9 for deaths before 1999 or ICD-10 codes I00YI78 for deaths occurring from 1999 to 2008	CV mortality	Highest vs. lowest	0.89 (0.77–1.03)	Age, BMI, RSBP, smoking, non-HDL-C
						The National Death Index	All-cause mortality	Highest vs. lowest	0.89 (0.82–0.97)	

CAD: coronary artery disease; CVD: cardiovascular disease; ASCVD: atherosclerotic cardiovascular disease; TyG: triglyceride and glucose index; WC: waist circumference; BMI: body mass index; FHCVD: family history of cardiovascular disease; T2D: type 2 diabetes; HTP: hypertension; LDL: low density lipoprotein; LDL-C: low-density lipoprotein cholesterol; HDL-C: high-density lipoprotein cholesterol; MI: myocardial infarction; RSBP: resting systolic blood pressure; SBP: systolic blood pressure; eGFR: estimated glomerular filtration rate; TC: total cholesterol; hs-CRP: high-sensitive C-reactive protein; HbA1c: Glycated hemoglobin A1c; CT: computed tomography; MRI: magnetic resonance imaging; CCTA: coronary computed tomographic angiography; ECG: electrocardiogram; ICD-10: International Classification of Diseases, 10th Clinical Modification; NA: not application

Only one study had an NOS score of 6 [42], which raised concerns about selection and outcome bias. The remaining studies were all of high quality, with a score higher than 7 (Additional file 1: Table S5). All studies adjusted age and sex, with adjustment for other confounders varying considerably.

### Association between the TyG index and incidence of CVD

#### CAD

**Fig. 2:**
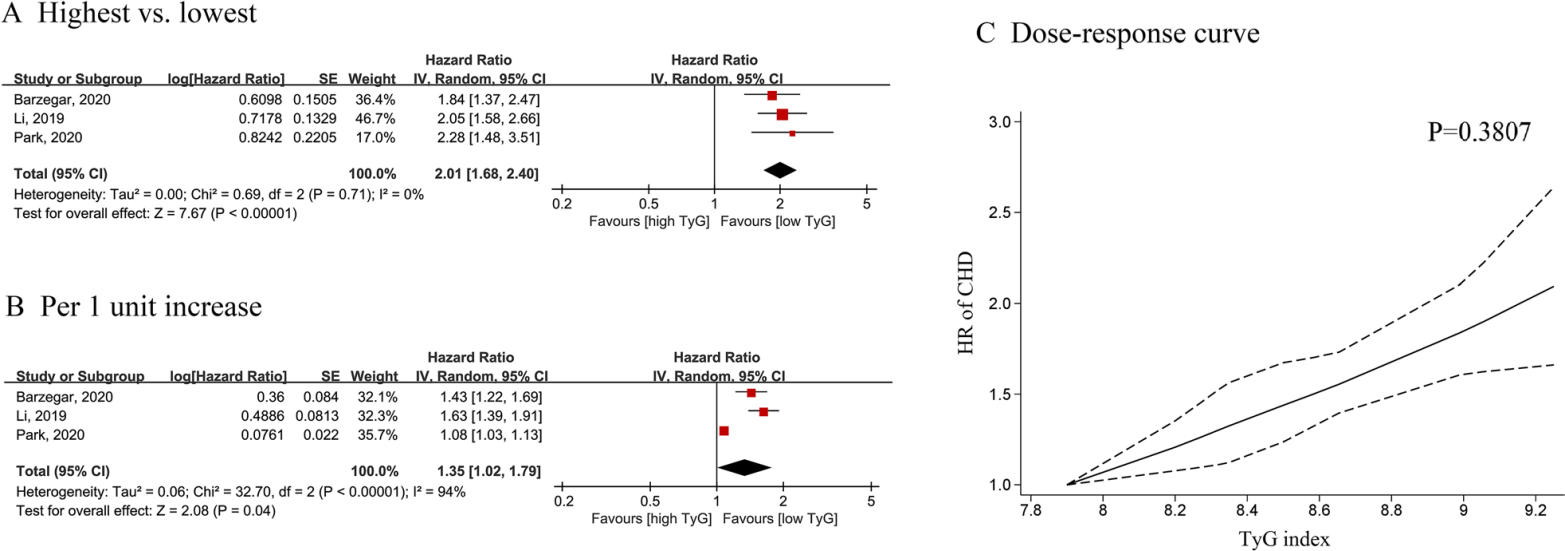
Forest plot and non-linear dose–response curve (**C**) for the association between TyG index and CAD in general population, analyzed as category variables (highest vs. lowest) (**A**) or continuous variables (per 1-unit increasement) (**B**). In the forest plot, the diamond indicates the pooled estimate. Red boxes are relative to study size and the black vertical lines indicate 95% CIs around the effect size estimate. The dose–response association were fitted by using restricted cubic spline regression model. The bold lines indicate the pooled restricted cubic spline model and the black dashed line indicates the 95% CIs of the pooled curve. TyG: Triglyceride-glucose; CAD: coronary artery disease

Three cohort studies including 30,054 participants were used for analysis of CAD and the TyG index [18, 37, 40]. Individuals in the general population with the highest or moderate TyG index had a higher risk of CAD (highest vs. lowest: HR = 2.01; 95% CI 1.68–2.40; I^2^ = 0%) (Fig. 2A) (moderate vs. lowest: HR = 1.45; 95% CI 1.19–1.76; I^2^ = 14%) compared to the lowest TyG index (Additional file 1: Figure S1A). The overall dose–response analysis results showed a 35% greater risk of stroke for every 1-unit increase in the TyG index (HR = 1.35; 95% CI 1.02–1.79, I^2^ = 94%) (Fig. 2B).

In addition, there was a positive, linear association between the TyG index and the risk of CAD (P_nonlinear_ = 0.3807) (Fig. 2C). Additional file 1: Table S6 shows the HR estimates for the linear exposure effects analysis of selected TyG index values, which were derived from linear figures.

#### MI

**Fig. 3:**
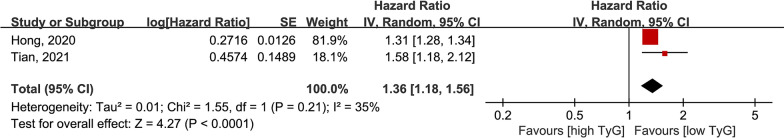
Forest plot for the association between TyG index and MI in general population, analyzed as category variables (highest vs. lowest). TyG: Triglyceride-glucose; MI: myocardial infarction

Analysis of two cohort studies with 5,614,862 individuals [20, 34] showed that a higher TyG index was related to a higher risk of MI in analysis of the highest vs. lowest TyG index groups (HR = 1.36; 95% CI 1.18–1.56; I^2^ = 35%) (Fig. 3) and moderate vs. lowest TyG index groups (HR = 1.17; 95% CI 1.14–1.19; I^2^ = 0%) (Additional file 1: Fig. S1B).

#### Composite of CVD incidence

**Fig. 4:**
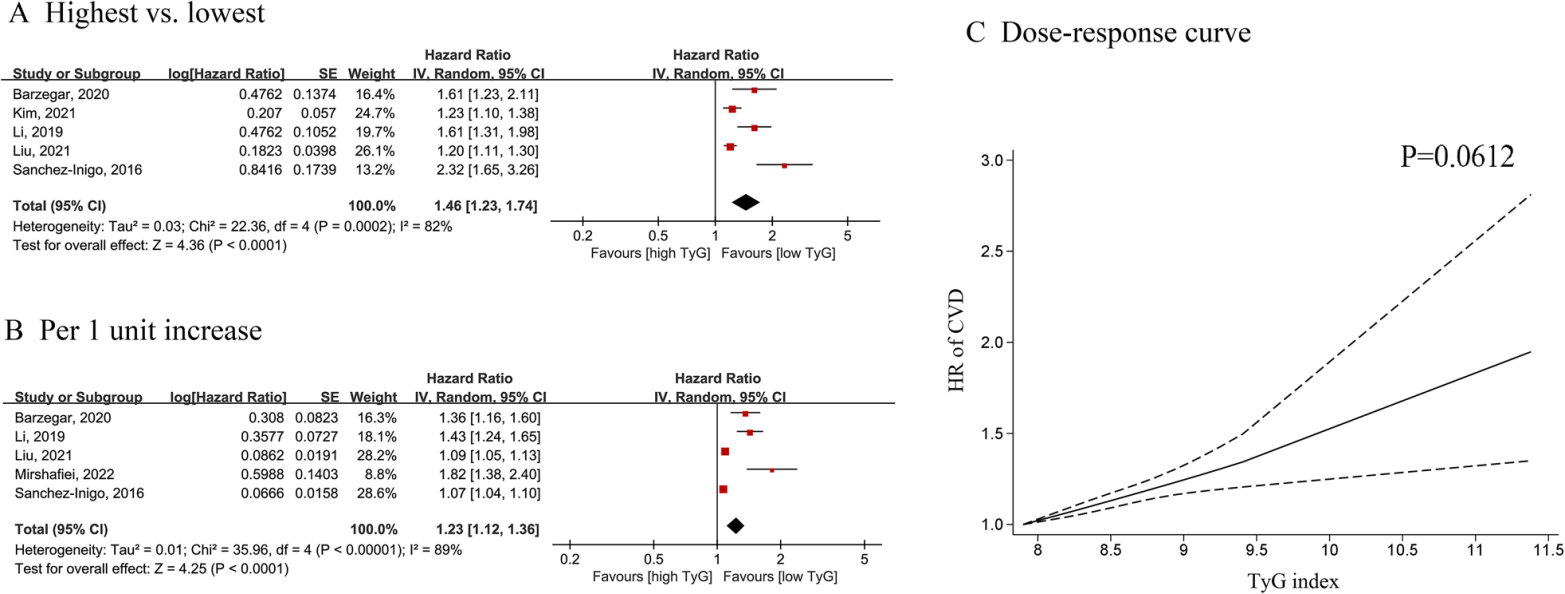
Forest plot and non-linear dose–response curve (**C**) for association between TyG and CVD in general population, analyzed as category variables (highest vs. lowest) (**A**) or continuous variables (per 1-unit increasement) (**B**). In the forest plot, the diamond indicates the pooled estimate. Red boxes are relative to study size and the black vertical lines indicate 95% CIs around the effect size estimate. The dose–response association were fitted by using restricted cubic spline regression model. The bold lines indicate the pooled restricted cubic spline model and the black dashed line indicates the 95% CIs of the pooled curve. TyG: Triglyceride-glucose; CVD: cardiovascular disease

Five cohorts were included for CVD analysis, with 259,757 participants. Pooled results showed that a higher TyG index was associated with an increased risk of CVD in analyses of highest vs. lowest (HR = 1.46; 95% CI 1.23–1.74; I2 = 82%) (Fig. 4A) or moderate vs. lowest (HR = 1.19; 95% CI 1.12–1.26; I^2^ = 0%) TyG index groups (Additional file 1: Fig. S1C). In dose–response analysis, the summary estimate revealed a 23% increased risk of CVD for a 1-unit increase in the TyG index (HR = 1.23, 95% CI 1.12–1.36, I^2^ = 89%) (Fig. 4B).

Curves also indicated a positive linear association of CVD with the TyG index (P_nonlinear_ = 0.0612) (Fig. 2C). Additional file 1: Table S6 provides HR estimates for the linear exposure effect analysis for the TyG index.

### Association between the TyG index and mortality

#### CV mortality

**Fig. 5:**
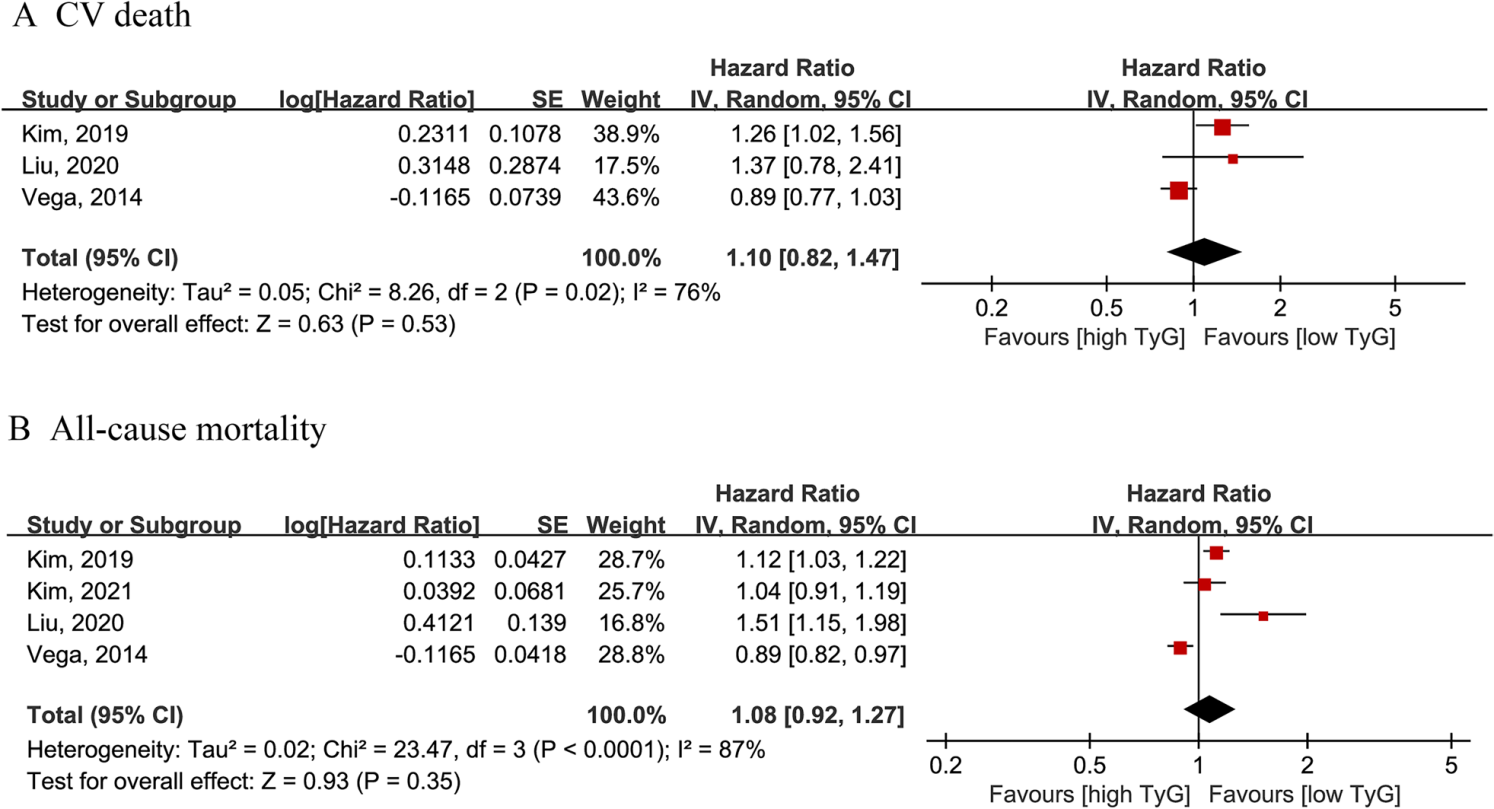
Forest plot for the association between TyG index and CV mortality (**A**) and all-cause mortality (**B**) in general population. TyG: Triglyceride-glucose; CV mortality: cardiovascular mortality

Three cohort studies with 377,091 participants [36, 38, 42] were included in the analysis between the TyG index and CV mortality. There was no significant association between a higher TyG index and the risk of CV mortality (highest vs. lowest: HR = 1.10; 95% CI 0.82–1.47), with high evidence of heterogeneity (I^2^ = 76%) (Fig. 5A).

#### All-cause mortality

We included four cohort studies with 521,694 participants [35, 36, 38, 42]. There was no statistically significant association between the TyG index and all-cause mortality (highest vs. lowest: HR = 1.08; 95% CI 0.92–1.27; I2 = 87% or moderate vs. lowest: HR = 0.99; 95% CI 0.81–1.21; I^2^ = 65%) (Fig. 3B and Additional file 1: Fig. S1D).

### Sensitivity analysis, subgroup analysis and publication bias

Sensitivity analysis by deleting one study showed consistent results (Additional file 1: Fig. S2). Due to the limited number of included studies (N < 10), subgroup and publication bias analyses were not performed according to the guidelines and predefined criteria.

### Quality assessment

We determined the quality of the evidence using GRADE. CVD and CAD outcomes were upgraded due to a dose–response relationship and the presence of a large effect for CAD. As there was greater heterogeneity in CVD (I2 = 82%) and mortality (CV mortality I^2^ = 76% and all-cause mortality I^2^ = 87%), and all outcomes not process analysis of publication bias, downgrades were given. Eventually, of the five outcomes included, GRADE assessment indicated very low certainty for CVD, MI, cardiovascular mortality and all-cause mortality, and moderate certainty for CAD (Additional file 1: Tables S7, S8).

## Discussion

In this meta-analysis, a higher TyG index was associated with an increased CAD, MI, and CVD incidence compared to a lower TyG index. In addition, a potential linear dose–response association was found between the TyG index and CAD/CVD incidence. We observed 35% and 23% increased risks of CAD and CVD, respectively, per 1-unit increase in the TyG index. However, a higher TyG index was not statistically associated with CV mortality or all-cause mortality. Taken together, these findings suggest that a higher TyG index may be associated with a higher risk of CVDs and that the index may be used as a risk factor independent of traditional clinical information for CVD incidence in the general population.

The previous “gold standard” indicator for measuring insulin resistance has been the HIEG clamp test [43], but application of this indicator is limited by its time-consuming and expensive procedure [44]. In recent years, other standards for measuring insulin sensitivity have been developed, such as the homeostasis model assessment-insulin resistance (HOMA-IR), quantitative insulin sensitivity check index (QUICKI), and triglyceride to high-density lipoprotein cholesterol (TG/HDL-C) ratio tools [9, 45]. Current evidence suggests that TyG is a reliable indicator of insulin resistance [23]. A study in Mexico demonstrated the high sensitivity (96.5%) and specificity (85.0%) of the TyG index for detecting insulin resistance compared to the gold standard (HIEG clamp test) [46]. Furthermore, TyG performed better than HOMA-IR in assessing insulin resistance (sensitivity 89%, specificity 67%) [47]. Both triglycerides and glucose are easily accessible and inexpensive to measure during hospitalization; thus, the TyG index does not seem to increase patients’ or overall health care costs. Based on our findings, the TyG index can be used as a reliable indicator for measuring insulin resistance and predicting the occurrence of CVD in the general population.

Several large prospective cohort studies have validated the positive relationship between the TyG index and hypertension [48–50], and a meta-analysis concluded that the TyG index can be used as a predictor of hypertension risk in the general adult population [51]. Ding et al. also reported that a higher TyG index may be associated with a higher incidence of atherosclerotic cardiovascular diseases (ASCVDs) in people without ASCVD at baseline [52]. To date, several community-based cohort studies have examined the relationship between the TyG index and various cardiovascular diseases. For example, in the Tehran Lipid and Glucose Study, Barzegar et al. demonstrated a greater risk of CAD and CVD with a higher TyG index [18]. Two large studies by Liu and Hong et al. also found a positive relationship between the TyG index and MI after following 96,541 and 5,593,134 individuals, respectively [19, 34]. The above studies provide evidence for the association of CVD with the TyG index in the general population.

Regarding the association between the TyG index and CV mortality and all-cause mortality in the general population, results were inconsistent across studies. For instance, Vega's study showed that the association between TyG and mortality risk disappeared after adjusting for age, BMI, resting systolic blood pressure, and smoking [42], though the analysis by Liu et al. showed a positive correlation [38]. Our results indicated no statistical association and these results must be interpreted with caution. In studies of other populations, most articles reported a positive correlation between the TyG index and CV mortality/all-cause mortality. For example, acute MI and coronary angiography patients with a high TyG index had a higher risk of CV mortality (RR = 2.71; 95% CI 1.92–3.83) and all-cause mortality (RR = 2.35; 95% CI 1.72–3.20) [53]. Guo’s study reported that a high TyG index was associated with CV mortality in patients with chronic heart failure and cases complicated by T2DM (RR = 4.42; 95% CI 1.49–13.15) [54]. Moreover, the positive relationship between the TyG index and all-cause mortality was detected in patients with chronic coronary syndrome or ischemic stroke [11, 55]. One prospective study including middle-aged Finnish men without diabetes showed that HOMA-IR was independently associated with death due to coronary artery disease (HR = 1.69; 95% CI 1.15–2.48), providing evidence for a positive association between insulin resistance and death and opening up the possibility of a relationship between the TyG index and death [56]. Overall, the limited number of included studies or insufficient follow-up time might have reduced the statistical power, and more prospective studies are needed to verify the relationship between the TyG index and mortality in the general population.

Sex may be a significant effect modifier. The I-Lan Longitudinal Aging Study (ILAS) showed a sex difference in the correlation between a high TyG index and subclinical atherosclerosis in patients without diabetes. Additionally, a significantly higher prevalence of subclinical atherosclerosis in the high TyG index group than in the low TyG index group (odd ratio [OR] = 1.510; 95% CI 1.010–2.257) was observed in women without diabetes but not in men without diabetes (OR = 0.827; 95% CI 1.556–1.231) [57]. This result may highlight the need for a sex-specific management strategy to prevent atherosclerosis. Of our included studies, adjustment for sex was not performed in two [35, 42]. Nonetheless, our results showed a higher TyG index to be associated with CAD/CVD, even after deleting sex-unadjusted studies (CVD HR = 1.59, 95% CI 1.21–2.09). However, the sex-specific association was not assessed due to limited studies, which need more research.

Diabetes is a vital confounding factor. Several previous studies have suggested that increased insulin resistance is associated with an increased risk of CV events in patients without diabetes [58, 59]. However, the relationship between insulin resistance and the risk of CV events in patients with diabetes remains controversial. In general, it is important to determine the relationship between the presence and severity of cardiovascular disease, such as CAD, in the general population based on the presence or absence of diabetes. One study found that although the prevalence of CAD (59.0% vs. 39.0%) and obstructive CAD (15.0% vs. 6.6%) was higher in patients with than without diabetes, the adjusted results showed that a high TyG index was associated with CAD (OR = 1.30; 95% CI 1.06–1.59) and obstructive CAD (OR = 1.86; 95% CI 1.23–2.81) only in the latter, with no association in the former (CAD OR = 1.16, 95% CI 0.75–1.80; obstructive CAD OR = 1.46, 95% CI 0.83–2.56) [60]. This finding suggests that insulin resistance significantly affects coronary atherosclerosis in patients without diabetes [61, 62], whereas in patients with diabetes, the primary mechanisms of atherosclerosis progression may be associated with other means [63, 64]. In addition, Si's article explored the association between the TyG index and CAD/T2DM, concluding that the TyG index is an risk factor for patients with coronary artery disease combined with T2DM and that it may be used as a clinical predictor of coronary artery disease combined with T2DM [65].

Regarding CVD outcomes, all included studies excluded patients with a history of CVD, and we showed a positive association between the TyG index and CVD incidence. Although diabetes is a strong risk factor for CVD, a few studies excluded a history of diabetes and CVD. In addition, one retrospective cohort study showed that the TyG index was associated with increased CVD incidence in a nondiabetes population after a median follow-up of 5.6 years [35]. Another prospective cohort study confirmed this association (per 1 SD HR = 1.82; 95% CI 1.38–2.40) after excluding patients with diabetes and CVD history after 6 years of follow-up [39]. Park demonstrated the relevance of CAD incidence for the TyG index in a nondiabetes population (HR = 2.28; 95% CI 1.69–2.40) [40]. However, our meta-analysis results showed no significant association between MI incidence and the TyG index in subgroup analysis of the population without diabetes (HR = 1.55, 95% CI 0.88–2.74, I2 = 98%) (Additional file 1: Figure S3) [20, 34]. Overall, before applying the TyG index for assessing CVD incidence and mortality, more studies based on individuals free of diabetes are needed to decrease reverse causality.

### Comparison with a previous study

Wang et al. found that the TyG index a positive association between TyG index and hypertension risk in the general adult population [51], and Li et al. demonstrated that the index can predict adverse events in patients with acute coronary syndrome [66]. Previous meta-analyses have also examined the relationship between the TyG index and the prognosis of patients with acute coronary syndromes [12, 66]. Compared to previous meta-analysis, the population in our study was the general population. In addition, our study included more types of cardiovascular diseases and mortality, and to produce more accurate results, the association between various cardiovascular diseases and the TyG index was analyzed separately.

### Potential mechanism

TyG may be a reliable indicator of insulin resistance [23], and a prior study demonstrated its validity as an alternative marker of insulin resistance [67], with high sensitivity (96.5%) and specificity (85.0%) [46]. Therefore, insulin resistance can be determined by measuring the TyG index and inferring the incidence of CVD. Insulin resistance is characterized by a low degree of systemic inflammation, which can lead to endothelial dysfunction [51]. At the same time, insulin resistance contributes to atherosclerosis and plaque progression through a variety of mechanisms, including changes in classical cardiovascular disease risk factors and downregulation of insulin signaling pathways [68].

Several risk factors that epitomize the development of cardiovascular disease are also closely related to TyG index results, such as coronary artery calcification (CAC), which was not included in our meta-analysis. Park's study (1175 participants followed up for 4.2 years) showed the TyG index to be an independent predictor of CAC progression (OR = 1.82, 95% CI 1.20–2.77) [69]. Furthermore, a Korean-based study found the TyG index to be better at predicting CAC (OR = 1.95, 95% CI 1.23–3.11) than HOMA-IR (OR = 1.64, 95% CI 1.12–2.40) [70]. Additionally, the development of CAC provides evidence for the development of CAD.

In estimates performed with the Archimedes Model, insulin resistance is an important cause of coronary artery disease [71], which may be assessed with the TyG index. A genetic study including 63,746 CAD patients and 130,681 healthy individuals reported that lipid metabolism and inflammation are key biological processes involved in the pathogenesis of coronary atherosclerosis, supporting the role of insulin resistance in the pathophysiology of CAD [72]. Insulin resistance in liver and adipose tissues drives the development of atherosclerotic dyslipidemia, generates a low-grade inflammatory state, and increases release of inflammatory markers [73]. It also affects blood pressure, endothelial cells, and macrophages [73].

### Limitations

Our meta-analysis has several limitations. First, this was a meta-analysis based on cohort studies. Despite the inclusion of studies with multivariate analysis, residual confounding factors may have influenced our results; thus, causation cannot be proven. Second, a total of 12 studies were included in our analysis, which is a relatively small sample size. Outcomes were inconsistent across the 12 studies we included, resulting in a small number of studies included in each outcome (MI = 2, CV death = 3). This may be another cause of bias and inaccurate results. Continued attention to relevant research and refinement of each analysis are needed in the future. In addition, of the 12 studies included, most were conducted in Asia, with only two conducted in Western countries (Europe and America). However, the limitation of the number of articles precluded us from examining regional variation, and future research should elucidate whether there are regional differences to demonstrate the association of the TyG index with CVD and mortality in the general population. Third, substantial heterogeneity was found, which might have derived from differences in TyG index measurement methods, study design, follow-up, diabetes status, and other clinical characteristics across studies. Fourth, the limited number of included studies prevented us from conducting subgroup analysis; hence, the potential influence of confounding and potential intermediate factors needs further investigation. Finally, limited studies excluded individuals with preexisting diabetes, and subgroup analysis stratified by diabetes was not available. Diabetes has been considered a main risk factor for CVD and mortality; therefore, application of the TyG index for CVD incidence can be easily biased by diabetes, and additional studies in nondiabetes populations are needed to reduce reverse causality before applying the TyG index to examine CVD incidence and mortality.

### Clinical implications

Although the TyG index is not directly applied in clinical guidelines, the role of glycemic and triglyceride control and diabetes management in the prevention of cardiovascular disease is reflected in some guidelines. Recently, the American Diabetes Association (ADA) published its 2022 diabetes criteria, which detail that patients with elevated triglyceride levels (≥ 150 mg/dL [1.7 mmol/L]) should implement enhanced lifestyle interventions and optimal glycemic control [74]. Control of blood glucose and triglycerides is effective in reducing the TyG index, an indicator of insulin resistance, which is one of the main triggers of T2DM. There is a strong association between these factors and clinical guidelines.

Our results show that an elevated TyG index is strongly associated with a high risk of CAD, MI and CVD, suggesting that the incidence of CVD can be significantly reduced by controlling TyG index-related factors or triggers that contribute to elevated TyG, such as blood glucose. As a simple-measurement indicator, the TyG index may be used more often in clinical practice. With respect to public health implications, early detection of CVD onset can be achieved by assessing the TyG index of the population, and prompt intervention after disease risk is identified may be effective in reducing the incidence of CVD in the general population. The TyG index might be added to routine health check-up programs to screen for the development of diabetes and cardiovascular disease. However, more research is needed before application of the TyG index to evaluate CVD incidence in the clinic.

Based on our study, the TyG index may be considered an independent predictor for CVD incidence in the general population. Further studies are required to investigate dose–response relationships and determine cutoff values. On the other hand, addition of the TyG index to the Framingham Risk Score (FRS) did not lead to improvement in its predictive power; thus, adding the TyG index to the FRS does not improve CVD risk prediction [18]. Further studies and more articles are needed to confirm the relevance of the TyG index to CVD, determine the predictive effect of the TyG index, and ascertain whether the TyG index can improve the predictive power of existing cardiovascular risk scores.

## Conclusion

In conclusion, based on the current evidence from observational studies, a high TyG index may have a significant relationship with the incidence of CAD, MI and CVD but not cardiovascular mortality or all-cause mortality in the general population. In addition, there is a potential positive linear relationship between the TyG index and CAD and the composite CVD incidence. GRADE assessment indicated very low certainty for CVD, MI, cardiovascular mortality and all-cause mortality, and moderate certainty for CAD. Considering the limited evidence from non-Asian populations and possible bias due to diabetes, further prospective studies in nondiabetes and non-Asian populations are needed to explore the association of the TyG index with CVD incidence and mortality.

## Supplementary Information

**Additional file 1. **Supplemental Tables and Figures.

## Data Availability

The datasets used and analyzed during the current study are available from the corresponding author on reasonable request.
