# Supplementary material for: Relationship between the triglyceride-glucose index and risk of cardiovascular diseases and mortality in the general population: a systematic review and meta-analysis
Source: Cardiovasc Diabetol. 2022 Jul 1;21:124. doi: 10.1186/s12933-022-01546-0 (PMC9250255; doi:10.1186/s12933-022-01546-0)
Supplement: Supplementary file 1 — Additional file 1. Supplemental Tables and Figures. [file 12933_2022_1546_MOESM1_ESM.docx]

**Table S1. PRISMA 2020 Checklist**

| **Section and Topic** | **Item #** | **Checklist item** | **Location where item is reported** |
| --- | --- | --- | --- |
| **TITLE** | | |  |
| Title | 1 | Identify the report as a systematic review. | 1 |
| **ABSTRACT** | | |  |
| Abstract | 2 | See the PRISMA 2020 for Abstracts checklist. | none |
| **INTRODUCTION** | | |  |
| Rationale | 3 | Describe the rationale for the review in the context of existing knowledge. | 5-6 |
| Objectives | 4 | Provide an explicit statement of the objective(s) or question(s) the review addresses. | 5-6 |
| **METHODS** | | |  |
| Eligibility criteria | 5 | Specify the inclusion and exclusion criteria for the review and how studies were grouped for the syntheses. | 3-4 |
| Information sources | 6 | Specify all databases, registers, websites, organisations, reference lists and other sources searched or consulted to identify studies. Specify the date when each source was last searched or consulted. | 6-8 |
| Search strategy | 7 | Present the full search strategies for all databases, registers and websites, including any filters and limits used. | 7 |
| Selection process | 8 | Specify the methods used to decide whether a study met the inclusion criteria of the review, including how many reviewers screened each record and each report retrieved, whether they worked independently, and if applicable, details of automation tools used in the process. | 7 |
| Data collection process | 9 | Specify the methods used to collect data from reports, including how many reviewers collected data from each report, whether they worked independently, any processes for obtaining or confirming data from study investigators, and if applicable, details of automation tools used in the process. | 7-8 |
| Data items | 10a | List and define all outcomes for which data were sought. Specify whether all results that were compatible with each outcome domain in each study were sought (e.g. for all measures, time points, analyses), and if not, the methods used to decide which results to collect. | 7-8 |
|  | 10b | List and define all other variables for which data were sought (e.g. participant and intervention characteristics, funding sources). Describe any assumptions made about any missing or unclear information. | 7-8 |
| Study risk of bias assessment | 11 | Specify the methods used to assess risk of bias in the included studies, including details of the tool(s) used, how many reviewers assessed each study and whether they worked independently, and if applicable, details of automation tools used in the process. | 7-8 |
| Effect measures | 12 | Specify for each outcome the effect measure(s) (e.g. risk ratio, mean difference) used in the synthesis or presentation of results. | 8 |
| Synthesis methods | 13a | Describe the processes used to decide which studies were eligible for each synthesis (e.g. tabulating the study intervention characteristics and comparing against the planned groups for each synthesis (item #5)). | 9 |
|  | 13b | Describe any methods required to prepare the data for presentation or synthesis, such as handling of missing summary statistics, or data conversions. | 9 |
|  | 13c | Describe any methods used to tabulate or visually display results of individual studies and syntheses. | 9 |
|  | 13d | Describe any methods used to synthesize results and provide a rationale for the choice(s). If meta-analysis was performed, describe the model(s), method(s) to identify the presence and extent of statistical heterogeneity, and software package(s) used. | 9-10 |
|  | 13e | Describe any methods used to explore possible causes of heterogeneity among study results (e.g. subgroup analysis, meta-regression). | 10 |
|  | 13f | Describe any sensitivity analyses conducted to assess robustness of the synthesized results. | 10 |
| Reporting bias assessment | 14 | Describe any methods used to assess risk of bias due to missing results in a synthesis (arising from reporting biases). | 10 |
| Certainty assessment | 15 | Describe any methods used to assess certainty (or confidence) in the body of evidence for an outcome. | 10 |
| **RESULTS** | | |  |
| Study selection | 16a | Describe the results of the search and selection process, from the number of records identified in the search to the number of studies included in the review, ideally using a flow diagram. | 10-11 |
|  | 16b | Cite studies that might appear to meet the inclusion criteria, but which were excluded, and explain why they were excluded. | 10-11 |
| Study characteristics | 17 | Cite each included study and present its characteristics. | 11 and table1 |
| Risk of bias in studies | 18 | Present assessments of risk of bias for each included study. | 12 |
| Results of individual studies | 19 | For all outcomes, present, for each study: (a) summary statistics for each group (where appropriate) and (b) an effect estimate and its precision (e.g. confidence/credible interval), ideally using structured tables or plots. | Table 1 |
| Results of syntheses | 20a | For each synthesis, briefly summarise the characteristics and risk of bias among contributing studies. | 11 |
|  | 20b | Present results of all statistical syntheses conducted. If meta-analysis was done, present for each the summary estimate and its precision (e.g. confidence/credible interval) and measures of statistical heterogeneity. If comparing groups, describe the direction of the effect. | 12-13 |
|  | 20c | Present results of all investigations of possible causes of heterogeneity among study results. | 12-13 |
|  | 20d | Present results of all sensitivity analyses conducted to assess the robustness of the synthesized results. | 8-10 |
| Reporting biases | 21 | Present assessments of risk of bias due to missing results (arising from reporting biases) for each synthesis assessed. | 14 |
| Certainty of evidence | 22 | Present assessments of certainty (or confidence) in the body of evidence for each outcome assessed. | 14-15 |
| **DISCUSSION** | | |  |
| Discussion | 23a | Provide a general interpretation of the results in the context of other evidence. | 18-19 |
|  | 23b | Discuss any limitations of the evidence included in the review. | 22-24 |
|  | 23c | Discuss any limitations of the review processes used. | 22-23 |
|  | 23d | Discuss implications of the results for practice, policy, and future research. | 23-24 |
| **OTHER INFORMATION** | | |  |
| Registration and protocol | 24a | Provide registration information for the review, including register name and registration number, or state that the review was not registered. | 6 |
|  | 24b | Indicate where the review protocol can be accessed, or state that a protocol was not prepared. | 6 |
|  | 24c | Describe and explain any amendments to information provided at registration or in the protocol. | none |
| Support | 25 | Describe sources of financial or non-financial support for the review, and the role of the funders or sponsors in the review. | 26 |
| Competing interests | 26 | Declare any competing interests of review authors. | 26 |
| Availability of data, code and other materials | 27 | Report which of the following are publicly available and where they can be found: template data collection forms; data extracted from included studies; data used for all analyses; analytic code; any other materials used in the review. | 26 |

*From:*  Page MJ, McKenzie JE, Bossuyt PM, Boutron I, Hoffmann TC, Mulrow CD, et al. The PRISMA 2020 statement: an updated guideline for reporting systematic reviews. BMJ 2021;372:n71. doi: 10.1136/bmj.n71

For more information, visit: <http://www.prisma-statement.org/>

**Table S2:** Detailed description of the search strategy

| **PubMed** | |
| --- | --- |
| #1 | ‘cardiovascular disease’ [MeSH Terms] |
| #2 | ‘cardiovascular disease’ OR ‘cardiovascular diseases’ OR ‘CVD’ OR ‘coronary heart disease’ OR ‘CHD’ OR ‘coronary artery disease’ OR ‘CAD’ OR ‘myocardial infarction’ OR ‘MI’ OR ‘coronary arteriosclerosis’ OR ‘heart attack’ OR ‘heart failure’ OR ‘HF’ OR ‘heart decompensation’ OR ‘atrial fibrillation’ OR ‘AF’ OR ‘sudden cardiac death’ OR ‘SCD’ OR ‘arrhythmia’ OR ‘cardiomyopathy’ OR ‘hypertrophic cardiomyopathy’ OR ‘HCM’ OR ‘dilated cardiomyopathy’ OR ‘DCM’ |
| #3 | ‘mortality’[MeSH Terms] |
| #4 | ‘mortality’ OR ‘death’ OR ‘cardiovascular death’ OR ‘all-cause mortality’ |
| #5 | ‘triglyceride-glucose index’[MeSH Terms] |
| #6 | ‘TyG index’ OR ‘triglyceride-glucose index’ |
| #7 | #1 OR #2 OR #3 OR #4 |
| #8 | #5 OR #6 |
| #9 | #7 AND #8 |
| **Embase** | |
| #1 | ‘cardiovascular disease’:ab,ti |
| #2 | ‘cardiovascular disease’ OR ‘cardiovascular diseases’ OR ‘CVD’ OR ‘coronary heart disease’ OR ‘CHD’ OR ‘coronary artery disease’ OR ‘CAD’ OR ‘myocardial infarction’ OR ‘MI’ OR ‘coronary arteriosclerosis’ OR ‘heart attack’ OR ‘heart failure’ OR ‘HF’ OR ‘heart decompensation’ OR ‘atrial fibrillation’ OR ‘AF’ OR ‘sudden cardiac death’ OR ‘SCD’ OR ‘arrhythmia’ OR ‘cardiomyopathy’ OR ‘hypertrophic cardiomyopathy’ OR ‘HCM’ OR ‘dilated cardiomyopathy’ OR ‘DCM’ |
| #3 | ‘mortality’:ab,ti |
| #4 | ‘mortality’ OR ‘death’ OR ‘cardiovascular death’ OR ‘all-cause mortality’ |
| #5 | ‘triglyceride-glucose index’:ab.ti |
| #6 | ‘TyG index’ OR ‘triglyceride-glucose index’ |
| #7 | #1 OR #2 OR #3 OR #4 |
| #8 | #5 OR #6 |
| #9 | #7 AND #8 |
| **Cochrane** | |
| #1 | ‘cardiovascular disease’:ti, ab, kw |
| #2 | ‘cardiovascular disease’ OR ‘cardiovascular diseases’ OR ‘CVD’ OR ‘coronary heart disease’ OR ‘CHD’ OR ‘coronary artery disease’ OR ‘CAD’ OR ‘myocardial infarction’ OR ‘MI’ OR ‘coronary arteriosclerosis’ OR ‘heart attack’ OR ‘heart failure’ OR ‘HF’ OR ‘heart decompensation’ OR ‘atrial fibrillation’ OR ‘AF’ OR ‘sudden cardiac death’ OR ‘SCD’ OR ‘arrhythmia’ OR ‘cardiomyopathy’ OR ‘hypertrophic cardiomyopathy’ OR ‘HCM’ OR ‘dilated cardiomyopathy’ OR ‘DCM’ |
| #3 | ‘mortality’ OR ‘cardiovascular death’ OR ‘all-cause mortality’:ti,ab,kw |
| #4 | ‘mortality’ OR ‘death’ OR ‘cardiovascular death’ OR ‘all-cause mortality’ |
| #5 | ‘triglyceride-glucose index’:ti, ab, kw |
| #6 | ‘TyG index’ OR ‘triglyceride-glucose index’ |
| #7 | #1 OR #2 OR #3 OR #4 |
| #8 | #5 OR #6 |
| #9 | #7 AND #8 |

**Table S3:** Studies excluded (n=72) with reasons

| Studies excluded | Reasons |
| --- | --- |
| Akbar, 2017 [1] | Not target population: type 2 diabetes mellitus |
| Alizargar, 2018 [2] | Not target outcome: hypertension |
| Banderali, 2014 [3] | Not target outcome: insulin resistance |
| Chen, 2020[4] | Not target outcome: new-onset diabetes |
| Cho, 2019[5] | Cross-sectional study |
| Chiu, 2020 [6] | Not target outcome: left ventricular dysfunction and atherosclerosis |
| Cho, 2021 [7] | Not target exposure: triglyceride glucose-waist circumference |
| Ding, 2021[8] | Meta analysis |
| Du, 2020 [9] | Not target exposure: triglyceride glucose-body mass index |
| Duran, 2020 [10] | Not target outcome: peripheral artery disease |
| Fiorentino, 2019 [11] | Not target outcome: insulin resistance |
| Guo, 2021[12] | Not target outcome: arterial stiffness |
| Gao, 2021 [13] | Not target outcome: peripheral artery disease |
| Hu, 2020 [14] | Not target population: patients with acute coronary syndrome undergoing percutaneous coronary intervention |
| Huang, 2021 [15] | Insufficient data |
| Irham, 2021 [16] | Not target population: acute coronary syndrome |
| Jin, 2018 [17] | Insufficient data |
| Jin, 2018 [18] | Not target exposure: triglyceride glucose and haemoglobin glycation index |
| Kim, 2017 [19] | Not target outcome: coronary artery calcification |
| Lee, 2016 [20] | Not target outcome: incident diabetes |
| Lee, 2016 [21] | Not target population: type 2 diabetes |
| Lee, 2021 [22] | Insufficient data |
| Lee, 2018 [23] | Not target outcome: arterial stiffness |
| Li, 2021 [24] | Not target population: acute coronary syndrome |
| Li, 2020 [25] | Not target population: hypertensive patients |
| Li, 2021 [26] | Insufficient data |
| Liu, 2020 [27] | Not target outcome: death |
| Lu, 2021 [28] | Not target exposure: gender difference |
| Luo, 2019 [29] | Not target population: acute ST-elevation myocardial infarction after percutaneous coronary intervention |
| Ma. 2020 [30] | Not target population: type 2 diabetes mellitus and acute coronary syndrome |
| Mao, 2019 [31] | Not target population: non-ST-segment elevation acute coronary syndrome |
| Morales-Gurrola, 2020 [32] | Not target outcome: metabolically obese normal-weight phenotype |
| Nam, 2020 [33] | Not target outcome: subclinical vertebral small vessel disease |
| Nam, 2021 [34] | Not target population: acute ischemic stroke |
| Nam, 2021 [35] | Abstract |
| Navarro-Gonzalez, 2016 [36] | Abstract |
| Neglia, 2021 [37] | Insufficient data |
| Nguyen, 2020 [38] | Abstract |
| Park, 2020 [39] | Insufficient data |
| Park, 2019 [40] | Not target outcome: coronary artery calcification |
| Park, 2020[41] | Cross-sectional study |
| Poon, 2020 [42] | Not target exposure: insulin resistance |
| Qin, 2020 [43] | Not target outcome: acute kidney injury |
| Rojas-Humpire, 2021[44] | Cross-sectional study |
| Salazar, 2018[45] | Cross-sectional study |
| Sanlialp, 2021 [46] | Letter |
| Sanlialp, 2021 [47] | Insufficient data |
| Si, 2021 [48] | Not target population: type 2 diabetes mellitus |
| Simental-Mendia, 2019 [49] | Not target population: children and adolescents |
| Su, 2019 [50] | Not target population: type 2 diabetes mellitus |
| Su, 2021 [51] | Not target outcome: arterial stiffness |
| Thai, 2020 [52] | Not target outcome: coronary artery stenosis |
| Tian, 2021 [53] | Insufficient data |
| Vega, 2014 [54] | Not target exposure: triglyceride-to-high-density-lipoprotein-cholesterol ratio |
| Wang, 2021 [55] | Not target outcome: intra- and extra- cranial arterial stenosis |
| Wang, 2020 [56] | Not target population: acute coronary syndrome |
| Wang, 2021 [57] | Insufficient data |
| Won, 2018 [58] | Cross-sectional study |
| Won, 2020 [59] | Not target outcome: coronary plaque volume change |
| Won, 2020 [60] | Not target outcome: coronary artery calcification |
| Won, 2018 [61] | Not target outcome: arterial stiffness |
| Wu, 2021 [62] | Not target population: non-normotensive population |
| Yan, 2019 [63] | Not target outcome: death |
| Yang, 2021 [64] | Not target population: undergoing percutaneous coronary intervention |
| Zhang, 2021 [65] | Not target outcome: hypertension |
| Zhang, 2021 [66] | Nor target population: type 2 diabetes mellitus and acute myocardial infarction |
| Zhang, 2021 [67] | Insufficient data |
| Zhao, 2021 [68] | Not target outcome: insulin resistance |
| Zhao, 2020 [69] | Not target population: non-ST-segment elevation acute coronary syndrome |
| Zhao, 2019 [70] | Not target outcome: macro- and microvascular damage |
| Zheng, 2017 [71] | Not target outcome: hypertension |
| Zhu, 2021 [72] | Not target population: acute coronary syndrome |

1. Akbar M, Bhandari U, Habib A, Ahmad R: **Potential Association of Triglyceride Glucose Index with Cardiac Autonomic Neuropathy in Type 2 Diabetes Mellitus Patients**. *J Korean Med Sci* 2017, **32**(7):1131-1138.

2. Alizargar J, Bai CH: **Comparison of Carotid Ultrasound Indices and the Triglyceride Glucose Index in Hypertensive and Normotensive Community-Dwelling Individuals: A Case Control Study for Evaluating Atherosclerosis**. *Medicina (Kaunas)* 2018, **54**(5).

3. Banderali G, Arrizza C, Salvioni M, Mariani B, Riva E, Verduci E: **Triglycerides/glucose index in obese children and adolescents**. *Archives of Disease in Childhood* 2014, **99**:A294-A295.

4. Chen CL, Liu L, Lo K, Huang JY, Yu YL, Huang YQ, Feng YQ: **Association Between Triglyceride Glucose Index and Risk of New-Onset Diabetes Among Chinese Adults: Findings From the China Health and Retirement Longitudinal Study**. *Front Cardiovasc Med* 2020, **7**:610322.

5. Cho YR, Ann SH, Won KB, Park GM, Kim YG, Yang DH, Kang JW, Lim TH, Kim HK, Choe J *et al*: **Association between insulin resistance, hyperglycemia, and coronary artery disease according to the presence of diabetes**. *Sci Rep* 2019, **9**(1):6129.

6. Chiu TH, Tsai HJ, Chiou HYC, Wu PY, Huang JC, Chen SC: **A high triglyceride–glucose index is associated with left ventricular dysfunction and atherosclerosis**. *International Journal of Medical Sciences* 2021, **18**(4):1051-1057.

7. Cho YK, Lee J, Kim HS, Kim EH, Lee MJ, Yang DH, Kang JW, Jung CH, Park JY, Kim HK *et al*: **Triglyceride glucose‐waist circumference better predicts coronary calcium progression compared with other indices of insulin resistance: a longitudinal observational study**. *Journal of Clinical Medicine* 2021, **10**(1):1-15.

8. Ding X, Wang X, Wu J, Zhang M, Cui M: **Triglyceride–glucose index and the incidence of atherosclerotic cardiovascular diseases: a meta-analysis of cohort studies**. *Cardiovascular Diabetology* 2021, **20**(1).

9. Du Z, Xing L, Lin M, Sun Y: **Estimate of prevalent ischemic stroke from triglyceride glucose-body mass index in the general population**. *BMC Cardiovasc Disord* 2020, **20**(1):483.

10. Duran Karaduman B, Ayhan H, Keleş T, Bozkurt E: **The triglyceride-glucose index predicts peripheral artery disease complexity**. *Turk J Med Sci* 2020, **50**(5):1217-1222.

11. Fiorentino TV, Marini MA, Succurro E, Andreozzi F, Sesti G: **Relationships of surrogate indexes of insulin resistance with insulin sensitivity assessed by euglycemic hyperinsulinemic clamp and subclinical vascular damage**. *BMJ Open Diabetes Res Care* 2019, **7**(1):e000911.

12. Guo W, Zhu W, Wu J, Li X, Lu J, Qin P, Zhu C, Xu N, Zhang Q: **Triglyceride Glucose Index Is Associated With Arterial Stiffness and 10-Year Cardiovascular Disease Risk in a Chinese Population**. *Front Cardiovasc Med* 2021, **8**:585776.

13. Gao JW, Hao QY, Gao M, Zhang K, Li XZ, Wang JF, Vuitton DA, Zhang SL, Liu PM: **Triglyceride-glucose index in the development of peripheral artery disease: findings from the Atherosclerosis Risk in Communities (ARIC) Study**. *Cardiovasc Diabetol* 2021, **20**(1):126.

14. Hu C, Zhang J, Liu J, Liu Y, Gao A, Zhu Y, Zhao Y: **Discordance between the triglyceride glucose index and fasting plasma glucose or HbA1C in patients with acute coronary syndrome undergoing percutaneous coronary intervention predicts cardiovascular events: a cohort study from China**. *Cardiovasc Diabetol* 2020, **19**(1):116.

15. Huang YC, Huang JC, Lin CI, Chien HH, Lin YY, Wang CL, Liang FW, Dai CY, Chuang HY: **Comparison of Innovative and Traditional Cardiometabolic Indices in Estimating Atherosclerotic Cardiovascular Disease Risk in Adults**. *Diagnostics (Basel)* 2021, **11**(4).

16. Irham A, Umar H, Adam FMS, Mappangara I, Bakri S, Halim R, Zainuddin A: **Concordance between triglyceride glucose index and admission insulin resistance index in non-diabetic subjects of acute coronary syndrome**. *Open Access Macedonian Journal of Medical Sciences* 2021, **9**:1044-1047.

17. Jin JL, Cao YX, Wu LG, You XD, Guo YL, Wu NQ, Zhu CG, Gao Y, Dong QT, Zhang HW *et al*: **Triglyceride glucose index for predicting cardiovascular outcomes in patients with coronary artery disease**. *J Thorac Dis* 2018, **10**(11):6137-6146.

18. Jin JL, Sun D, Cao YX, Guo YL, Wu NQ, Zhu CG, Gao Y, Dong QT, Zhang HW, Liu G *et al*: **Triglyceride glucose and haemoglobin glycation index for predicting outcomes in diabetes patients with new-onset, stable coronary artery disease: a nested case-control study**. *Ann Med* 2018, **50**(7):576-586.

19. Kim MK, Ahn CW, Kang S, Nam JS, Kim KR, Park JS: **Relationship between the triglyceride glucose index and coronary artery calcification in Korean adults**. *Cardiovasc Diabetol* 2017, **16**(1):108.

20. Lee DY, Lee ES, Kim JH, Park SE, Park CY, Oh KW, Park SW, Rhee EJ, Lee WY: **Predictive value of triglyceride glucose index for the risk of incident diabetes: A 4-year retrospective longitudinal study**. *PLoS ONE* 2016, **11**(9).

21. Lee EY, Yang HK, Lee J, Kang B, Yang Y, Lee SH, Ko SH, Ahn YB, Cha BY, Yoon KH *et al*: **Triglyceride glucose index, a marker of insulin resistance, is associated with coronary artery stenosis in asymptomatic subjects with type 2 diabetes**. *Lipids Health Dis* 2016, **15**(1):155.

22. Lee KY, Oh D, Yang EJ, Park HC, Jhee JH: **Association the triglyceride-glucose (TYG) index and coronary artery calcification progression in non-diabetic chronic kidney disease**. *Nephrology* 2021, **26**(SUPPL 1):34-35.

23. Lee SB, Ahn CW, Lee BK, Kang S, Nam JS, You JH, Kim MJ, Kim MK, Park JS: **Association between triglyceride glucose index and arterial stiffness in Korean adults**. *Cardiovasc Diabetol* 2018, **17**(1):41.

24. Li J, Ren L, Chang C, Luo L: **Triglyceride-Glukose Index Predicts Adverse Events in Patients with Acute Coronary Syndrome: A Meta-Analysis of Cohort Studies**. *Hormone and Metabolic Research* 2021.

25. Li M, Zhan A, Huang X, Hu L, Zhou W, Wang T, Zhu L, Bao H, Cheng X: **Positive association between triglyceride glucose index and arterial stiffness in hypertensive patients: the China H-type Hypertension Registry Study**. *Cardiovasc Diabetol* 2020, **19**(1):139.

26. Li Y, You A, Tomlinson B, Yue L, Zhao K, Fan H, Zheng L: **Insulin resistance surrogates predict hypertension plus hyperuricemia**. *Journal of Diabetes Investigation* 2021.

27. Liu XC, He GD, Lo K, Huang YQ, Feng YQ: **The Triglyceride-Glucose Index, an Insulin Resistance Marker, Was Non-linear Associated With All-Cause and Cardiovascular Mortality in the General Population**. *Front Cardiovasc Med* 2020, **7**:628109.

28. Lu YW, Chang CC, Chou RH, Tsai YL, Liu LK, Chen LK, Huang PH, Lin SJ: **Gender difference in the association between TyG index and subclinical atherosclerosis: results from the I-Lan Longitudinal Aging Study**. *Cardiovasc Diabetol* 2021, **20**(1):206.

29. Luo E, Wang D, Yan G, Qiao Y, Liu B, Hou J, Tang C: **High triglyceride-glucose index is associated with poor prognosis in patients with acute ST-elevation myocardial infarction after percutaneous coronary intervention**. *Cardiovasc Diabetol* 2019, **18**(1):150.

30. Ma X, Dong L, Shao Q, Cheng Y, Lv S, Sun Y, Shen H, Wang Z, Zhou Y, Liu X: **Triglyceride glucose index for predicting cardiovascular outcomes after percutaneous coronary intervention in patients with type 2 diabetes mellitus and acute coronary syndrome**. *Cardiovasc Diabetol* 2020, **19**(1):31.

31. Mao Q, Zhou D, Li Y, Wang Y, Xu SC, Zhao XH: **The Triglyceride-Glucose Index Predicts Coronary Artery Disease Severity and Cardiovascular Outcomes in Patients with Non-ST-Segment Elevation Acute Coronary Syndrome**. *Dis Markers* 2019, **2019**:6891537.

32. Morales-Gurrola G, Simental-Mendía LE, Castellanos-Juárez FX, Salas-Pacheco JM, Guerrero-Romero F: **The triglycerides and glucose index is associated with cardiovascular risk factors in metabolically obese normal-weight subjects**. *J Endocrinol Invest* 2020, **43**(7):995-1000.

33. Nam KW, Kwon HM, Jeong HY, Park JH, Kwon H, Jeong SM: **High triglyceride-glucose index is associated with subclinical cerebral small vessel disease in a healthy population: a cross-sectional study**. *Cardiovasc Diabetol* 2020, **19**(1):53.

34. Nam KW, Kwon HM, Lee YS: **High triglyceride-glucose index is associated with early recurrent ischemic lesion in acute ischemic stroke**. *Sci Rep* 2021, **11**(1):15335.

35. Nam KW, Kwon HM, Lee YS: **Triglyceride-glucose index on early recurrent ischemic lesion in acute ischemic stroke**. *European Stroke Journal* 2021, **6**(1 SUPPL):341-342.

36. Navarro-González D, Sánchez-Íñigo L, Fernández-Montero A, Pastrana-Delgado J, Alfredo Martínez J: **Risk of incident cardiovascular disease according to the metabolic health and obesity states**. *Cardiology (Switzerland)* 2016, **134**:393.

37. Neglia D, Aimo A, Caselli C, Gimelli A: **Triglycerides glucose index and high-sensitivity C-reactive protein are predictors of outcome in chronic coronary syndrome**. *European Heart Journal Cardiovascular Imaging* 2021, **22**(SUPPL 3):iii55.

38. Nguyen MT, Sultan A, Cosson E, Avignon A, Valensi P: **Relationship between triglycerides-glucose index and silent coronary artery disease in asymptomatic patients with type 2 diabetes**. *Diabetologia* 2020, **63**(SUPPL 1):S428-S429.

39. Park B, Lee YJ, Lee HS, Jung DH: **The triglyceride-glucose index predicts ischemic heart disease risk in Koreans: a prospective study using National Health Insurance Service data**. *Cardiovasc Diabetol* 2020, **19**(1):210.

40. Park K, Ahn CW, Lee SB, Kang S, Nam JS, Lee BK, Kim JH, Park JS: **Elevated TyG Index Predicts Progression of Coronary Artery Calcification**. *Diabetes Care* 2019, **42**(8):1569-1573.

41. Park GM, Cho YR, Won KB, Yang YJ, Park S, Ann SH, Kim YG, Park EJ, Kim SJ, Lee SG *et al*: **Triglyceride glucose index is a useful marker for predicting subclinical coronary artery disease in the absence of traditional risk factors**. *Lipids Health Dis* 2020, **19**(1):7.

42. Poon AK, Meyer ML, Tanaka H, Selvin E, Pankow J, Zeng D, Loehr L, Knowles JW, Rosamond W, Heiss G: **Association of insulin resistance, from mid-life to late-life, with aortic stiffness in late-life: the Atherosclerosis Risk in Communities Study**. *Cardiovasc Diabetol* 2020, **19**(1):11.

43. Qin Y, Tang H, Yan G, Wang D, Qiao Y, Luo E, Hou J, Tang C: **A High Triglyceride-Glucose Index Is Associated With Contrast-Induced Acute Kidney Injury in Chinese Patients With Type 2 Diabetes Mellitus**. *Front Endocrinol (Lausanne)* 2020, **11**:522883.

44. Rojas-Humpire R, Olarte-Durand M, Medina-Ramirez S, Gutierrez-Ajalcriña R, Canaza JF, Huancahuire-Vega S: **Insulin Resistance Indexes as Biomarkers of Lifetime Cardiovascular Risk among Adults from Peru**. *J Nutr Metab* 2021, **2021**:6633700.

45. Salazar J, Bermúdez V, Olivar LC, Torres W, Palmar J, Añez R, Ordoñez MG, Rivas JR, Martínez MS, Hernández JD *et al*: **Insulin resistance indices and coronary risk in adults from Maracaibo city, Venezuela: A cross sectional study**. *F1000Res* 2018, **7**:44.

46. Sanlialp SC: **Points to Be Resolved Regarding the Use of the Triglyceride Glucose Index for Cardiovascular Disease**. *Angiology* 2021.

47. Şanlialp SC, Nar G, Şen G, Günver MG, Şanlialp M: **High triglyceride glucose index does not show the presence and severity of coronary artery disease: A single- center study**. *E Journal of Cardiovascular Medicine* 2021, **9**(2):76-82.

48. Si Y, Fan W, Shan W, Zhang Y, Liu J, Han C, Sun L: **Association between triglyceride glucose index and coronary artery disease with type 2 diabetes mellitus in middle-aged and elderly people**. *Medicine (Baltimore)* 2021, **100**(9):e25025.

49. Simental-Mendía LE, Hernández-Ronquillo G, Gamboa-Gómez CI, Gómez-Díaz R, Rodríguez-Morán M, Guerrero-Romero F: **The triglycerides and glucose index is associated with elevated blood pressure in apparently healthy children and adolescents**. *Eur J Pediatr* 2019, **178**(7):1069-1074.

50. Su WY, Chen SC, Huang YT, Huang JC, Wu PY, Hsu WH, Lee MY: **Comparison of the effects of fasting glucose, hemoglobin a1c, and triglyceride–glucose index on cardiovascular events in type 2 diabetes mellitus**. *Nutrients* 2019, **11**(11).

51. Su Y, Wang S, Sun J, Zhang Y, Ma S, Li M, Zhang A, Cheng B, Cai S, Bao Q *et al*: **Triglyceride Glucose Index Associated With Arterial Stiffness in Chinese Community-Dwelling Elderly**. *Front Cardiovasc Med* 2021, **8**:737899.

52. Thai PV, Tien HA, Van Minh H, Valensi P: **Triglyceride glucose index for the detection of asymptomatic coronary artery stenosis in patients with type 2 diabetes**. *Cardiovasc Diabetol* 2020, **19**(1):137.

53. Tian X, Zuo Y, Chen S, Liu Q, Tao B, Wu S, Wang A: **Triglyceride–glucose index is associated with the risk of myocardial infarction: an 11-year prospective study in the Kailuan cohort**. *Cardiovascular Diabetology* 2021, **20**(1).

54. Vega GL, Barlow CE, Grundy SM, Leonard D, DeFina LF: **Triglyceride-to-high-density-lipoprotein-cholesterol ratio is an index of heart disease mortality and of incidence of type 2 diabetes mellitus in men**. *J Investig Med* 2014, **62**(2):345-349.

55. Wang A, Tian X, Zuo Y, Chen S, Zhang X, Guo J, Wu S, Zhao X: **Association of triglyceride–glucose index with intra- and extra-cranial arterial stenosis: a combined cross-sectional and longitudinal analysis**. *Endocrine* 2021.

56. Wang L, Cong HL, Zhang JX, Hu YC, Wei A, Zhang YY, Yang H, Ren LB, Qi W, Li WY *et al*: **Triglyceride-glucose index predicts adverse cardiovascular events in patients with diabetes and acute coronary syndrome**. *Cardiovasc Diabetol* 2020, **19**(1):80.

57. Wang S, Shi J, Peng Y, Fang Q, Mu Q, Gu W, Hong J, Zhang Y, Wang W: **Stronger association of triglyceride glucose index than the HOMA-IR with arterial stiffness in patients with type 2 diabetes: a real-world single-centre study**. *Cardiovasc Diabetol* 2021, **20**(1):82.

58. Won KB, Kim YS, Lee BK, Heo R, Han D, Lee JH, Lee SE, Sung JM, Cho I, Park HB *et al*: **The relationship of insulin resistance estimated by triglyceride glucose index and coronary plaque characteristics**. *Medicine (Baltimore)* 2018, **97**(21):e10726.

59. Won KB, Lee BK, Park HB, Heo R, Lee SE, Rizvi A, Lin FY, Kumar A, Hadamitzky M, Kim YJ *et al*: **Quantitative assessment of coronary plaque volume change related to triglyceride glucose index: The Progression of AtheRosclerotic PlAque DetermIned by Computed TomoGraphic Angiography IMaging (PARADIGM) registry**. *Cardiovasc Diabetol* 2020, **19**(1):113.

60. Won KB, Park EJ, Han D, Lee JH, Choi SY, Chun EJ, Park SH, Han HW, Sung J, Jung HO *et al*: **Triglyceride glucose index is an independent predictor for the progression of coronary artery calcification in the absence of heavy coronary artery calcification at baseline**. *Cardiovasc Diabetol* 2020, **19**(1):34.

61. Won KB, Park GM, Lee SE, Cho IJ, Kim HC, Lee BK, Chang HJ: **Relationship of insulin resistance estimated by triglyceride glucose index to arterial stiffness**. *Lipids Health Dis* 2018, **17**(1):268.

62. Wu Z, Zhou D, Liu Y, Li Z, Wang J, Han Z, Miao X, Liu X, Li X, Wang W *et al*: **Association of TyG index and TG/HDL-C ratio with arterial stiffness progression in a non-normotensive population**. *Cardiovascular Diabetology* 2021, **20**(1).

63. Yan Z, Yu D, Cai Y, Shang J, Qin R, Xiao J, Zhao B, Zhao Z, Simmons D: **Triglyceride Glucose Index Predicting Cardiovascular Mortality in Chinese Initiating Peritoneal Dialysis: A Cohort Study**. *Kidney Blood Press Res* 2019, **44**(4):669-678.

64. Yang J, Tang YD, Zheng Y, Li C, Zhou Q, Gao J, Meng X, Zhang K, Wang W, Shao C: **The Impact of the Triglyceride-Glucose Index on Poor Prognosis in NonDiabetic Patients Undergoing Percutaneous Coronary Intervention**. *Front Endocrinol (Lausanne)* 2021, **12**:710240.

65. Zhang F, Zhang Y, Guo Z, Yang H, Ren M, Xing X, Cong H: **The association of triglyceride and glucose index, and triglyceride to high-density lipoprotein cholesterol ratio with prehypertension and hypertension in normoglycemic subjects: A large cross-sectional population study**. *J Clin Hypertens (Greenwich)* 2021, **23**(7):1405-1412.

66. Zhang Y, Ding X, Hua B, Liu Q, Gao H, Chen H, Zhao XQ, Li W, Li H: **Predictive effect of triglyceride‑glucose index on clinical events in patients with type 2 diabetes mellitus and acute myocardial infarction: results from an observational cohort study in China**. *Cardiovasc Diabetol* 2021, **20**(1):43.

67. Zhang Y, Ren L, Ren M, Yang H, Li K, Cong H, Guo Z: **Correlation between the triglyceride–glucose index and high risk of cardiovascular disease: A cohort study of 102,061 subjects from Tianjin, China**. *Risk Management and Healthcare Policy* 2021, **14**:2803-2810.

68. Zhao Q, Cheng YJ, Xu YK, Zhao ZW, Liu C, Sun TN, Zhou YJ: **Comparison of various insulin resistance surrogates on prognostic prediction and stratification following percutaneous coronary intervention in patients with and without type 2 diabetes mellitus**. *Cardiovasc Diabetol* 2021, **20**(1):190.

69. Zhao Q, Zhang TY, Cheng YJ, Ma Y, Xu YK, Yang JQ, Zhou YJ: **Impacts of triglyceride-glucose index on prognosis of patients with type 2 diabetes mellitus and non-ST-segment elevation acute coronary syndrome: results from an observational cohort study in China**. *Cardiovasc Diabetol* 2020, **19**(1):108.

70. Zhao S, Yu S, Chi C, Fan X, Tang J, Ji H, Teliewubai J, Zhang Y, Xu Y: **Association between macro- and microvascular damage and the triglyceride glucose index in community-dwelling elderly individuals: the Northern Shanghai Study**. *Cardiovasc Diabetol* 2019, **18**(1):95.

71. Zheng R, Mao Y: **Triglyceride and glucose (TyG) index as a predictor of incident hypertension: a 9-year longitudinal population-based study**. *Lipids Health Dis* 2017, **16**(1):175.

72. Zhu Y, Liu K, Chen M, Liu Y, Gao A, Hu C, Li H, Zhu H, Han H, Zhang J *et al*: **Triglyceride-glucose index is associated with in-stent restenosis in patients with acute coronary syndrome after percutaneous coronary intervention with drug-eluting stents**. *Cardiovasc Diabetol* 2021, **20**(1):137.

**Table S4.** The definition of composite cardiovascular disease

| Study | Definition |
| --- | --- |
| Barzegar, 2020 | A composite of CHD plus fatal and non-fatal stroke. |
| Kim, 2021 | ICD-10 I20-I25 |
| Li, 2019 | Consisting of fatal and non-fatal CHD events and fatal and non-fatal cerebrovascular disease events |
| Liu, 2021 | MI and stroke |
| Mirshafiei, 2021 | NA |
| Sanchez-Inigo, 2016 | ICD-10 I20-I25, I63-I66, I73.9-I74 |

Abbreviation:

CVD: cardiovascular disease; CHD: coronary heart disease; ICD-10: International Classification of Diseases, 10th Clinical Modification.

**Table S5**. Quality assessment of included studies

| Author  (Publication Year) | Newcastle-Ottawa Scale | | | | | | | | | |
| --- | --- | --- | --- | --- | --- | --- | --- | --- | --- | --- |
|  | Selection | | | Comparability | | | Outcome | | | Total |
|  | a | b | c | d | e | f | g | h | i |  |
| Barzegar, 2020 | 1 | 1 | 1 | 1 | 1 | 1 | 1 | 1 | 1 | 9 |
| Hong, 2020 | 1 | 1 | 1 | 1 | 1 | 1 | 1 | 1 | 1 | 9 |
| Kim, 2019 | 1 | 1 | 1 | 1 | 1 | 1 | 0 | 1 | 1 | 8 |
| Kim, 2021 | 1 | 1 | 1 | 1 | 1 | 1 | 1 | 1 | 1 | 9 |
| Li, 2019 | 1 | 1 | 1 | 1 | 1 | 1 | 1 | 1 | 1 | 9 |
| Liu, 2020 | 1 | 1 | 1 | 1 | 1 | 1 | 0 | 1 | 1 | 8 |
| Liu, 2021 | 1 | 1 | 1 | 1 | 1 | 1 | 1 | 1 | 1 | 9 |
| Mirshafiei, 2021 | 1 | 1 | 1 | 1 | 1 | 1 | 1 | 1 | 1 | 9 |
| Park, 2020 | 1 | 1 | 1 | 1 | 1 | 1 | 1 | 0 | 0 | 7 |
| Sanchez-Inigo, 2016 | 1 | 1 | 1 | 1 | 1 | 1 | 1 | 1 | 1 | 9 |
| Tian, 2021 | 1 | 1 | 1 | 1 | 1 | 1 | 1 | 1 | 1 | 9 |
| Vega, 2014 | 0 | 0 | 1 | 1 | 1 | 1 | 0 | 1 | 1 | 6 |

1. Representativeness of the exposed cohort.
2. Selection of the non-exposed cohort.
3. Ascertainment of exposure.
4. Demonstration that outcome of interest was not present at start of study.
5. Comparability of cohorts on the basis of the design or analysis (adjusted for age).
6. Comparability of cohorts on the basis of the design or analysis (adjusted for any other factor).
7. Assessment of outcome.
8. Was follow-up long enough for outcomes to occur (>5 years).
9. Adequacy of follow-up of cohorts (>5 years).

**Table S6.** Tables of HRs and 95% CIs from non-linear dose-response analysis of TyG index and CAD/CVD

|  | CAD |
| --- | --- |
| TyG index | HR (95% CI) |
| 7.40 | 1 |
| 7.82 | 1.33 (1.20-1.48) |
| 8.01 | 1.43 (1.24-1.64) |
| 8.50 | 1.50 (1.22-1.83) |
| 8.82 | 1.51 (1.16-1.96) |
| 9.81 | 1.53 (1.02-2.30) |
|  |  |
|  | CAD |
| TyG index | HR (95% CI) |
| 7.40 | 1 |
| 7.80 | 1.08 (1.04-1.12) |
| 8.01 | 1.12 (1.07-1.18) |
| 8.30 | 1.19 (1.11-1.28) |
| 8.50 | 1.24 (1.14-1.35) |
| 8.71 | 1.30 (1.17-1.44) |
| 8.91 | 1.35 (1.19-1.54) |
| 10.89 | 2.03 (1.38-2.99) |

Abbreviation:

TyG: Triglyceride-Glucose Index; HR: hazard ratio; CI: confidence interval; CVD: cardiovascular disease; CAD: coronary artery disease.

**Table S7.** **Summary of findings for the cardiovascular diseases and mortality.**

| **TyG index and cardiovascular diseases/mortality** | | | | | | |
| --- | --- | --- | --- | --- | --- | --- |
| **Patient or population:** General population **Settings:** cardiovascular diseases incidence and mortality **Exposure:** TyG index (highest vs lowest) | | | | | | |
| **Outcomes** | **Illustrative comparative risks* (95% CI)** | | **Relative effect (95% CI)** | **No of Participants (studies)** | **Quality of the evidence (GRADE)** | **Comments** |
|  | Assumed risk | Corresponding risk |  |  |  |  |
|  | **Control** | **Outcome** |  |  |  |  |
| **CVD** ICD-10 Follow-up: 5.52-16.1 years | **Study population** | | **HR 1.46**  (1.23 to 1.74) | 259,757 (3 prospective and 2 retrospective cohort studies) | ⊕⊝⊝⊝ **very low**^1,4,7^ | There is a positive linear dose-response curve  I^2^=82%  No analysis of publication bias |
|  | **47 per 1000** | **68 per 1000** (58 to 81) |  |  |  |  |
| **CAD** ICD-10 Follow-up: 4.17-16.1 years | **Study population** | | **HR 2.01**  (1.68 to 2.4) | 30,054 (2 prospective and 1 retrospective cohort studies) | ⊕⊕⊕⊝ **moderate**^2,3,7^ | There is a positive linear dose-response curve  Large effect size (HR=2.01)  No analysis of publication bias |
|  | **20 per 1000** | **39 per 1000** (33 to 47) |  |  |  |  |
| **MI** ICD-10 Follow-up: 8.2-11.03 years | **Study population** | | **HR 1.36**  (1.18 to 1.56) | 5,614,862 (1 prospective and 1 retrospective cohort studies) | ⊕⊝⊝⊝ **very low**^7^ | No analysis of publication bias |
|  | **11 per 1000** | **16 per 1000** (14 to 18) |  |  |  |  |
| **CV mortality** ICD-10 or ICD-9 Follow-up: 5.66-14.75 years | **Study population** | | **HR 1.10**  (0.82 to 1.47) | 377,091 (3 respective cohort studies) | ⊝⊝⊝⊝ **very low**^5,7^ | Great heterogeneity (I^2^=76%)  No analysis of publication bias |
|  | **11 per 1000** | **12 per 1000** (9 to 16) |  |  |  |  |
| **all-cause mortality** database Follow-up: 5.66-14.75 years | **Study population** | | **HR 1.08**  (0.92 to 1.27) | 521,694 (4 retrospective cohort studies) | ⊝⊝⊝⊝ **very low**^6.7^ | Great heterogeneity (I^2^=87%)  No analysis of publication bias |
|  | **49 per 1000** | **53 per 1000** (45 to 62) |  |  |  |  |
| *The basis for the **assumed risk** (e.g. the median control group risk across studies) is provided in footnotes. The **corresponding risk** (and its 95% confidence interval) is based on the assumed risk in the comparison group and the **relative effect** of the intervention (and its 95% CI). **CI:** Confidence interval; **HR:** Hazard ratio; **CVD**: cardiovascular disease; **CAD**: coronary artery disease; **MI**: myocardial infarction; **TyG index**: triglyceride and glucose index; **CV** **mortality**: cardiovascular mortality; **ICD**: International Classification of Diseases | | | | | | |
| GRADE Working Group grades of evidence **High quality:** Further research is very unlikely to change our confidence in the estimate of effect.  **Moderate quality:** Further research is likely to have an important impact on our confidence in the estimate of effect and may change the estimate. **Low quality:** Further research is very likely to have an important impact on our confidence in the estimate of effect and is likely to change the estimate. **Very low quality:** We are very uncertain about the estimate. | | | | | | |
| ^1^ linear positive association; ^2^ HR=2.01; ^3^ linear positive association; ^4^ I^2^=82%; ^5^ I^2^=76%; ^6^ I^2^=87%; ^7^ No analysis of publication bias. | | | | | | |

**Table S8. GRADE evidence profile for the cardiovascular diseases and mortality.**

| **Quality assessment** | | | | | | | **No of patients** | | **Effect** | | **Quality** | **Importance** |  |
| --- | --- | --- | --- | --- | --- | --- | --- | --- | --- | --- | --- | --- | --- |
|  |  |  |  |  |  |  |  |  |  |  |  |  |  |
| **No of studies** | **Design** | **Risk of bias** | **Inconsistency** | **Indirectness** | **Imprecision** | **Other considerations** | **Outcome** | **Control** | **Relative (95% CI)** | **Absolute** |  |  |  |
| **CVD (follow-up 5.52-16.1 years; assessed with: ICD-10)** | | | | | | | | | | | | |  |
| 5 | observational studies | serious^1^ | no serious inconsistency | no serious indirectness | no serious imprecision | dose response gradient^2^ | 2604/26768  (9.7%) | 1255/26562  (4.7%) | HR 1.46 (1.23 to 1.74) | 21 more per 1000 (from 11 more to 34 more) | ⊕OOO VERY LOW | CRITICAL |  |
| **CAD (follow-up 4.17-16.1 years; assessed with: ICD-10)** | | | | | | | | | | | | |  |
| 3 | observational studies | serious^1^ | serious^3^ | no serious indirectness | no serious imprecision | strong association^4^ dose response gradient^1^ | 343/5619  (6.1%) | 112/5663  (2%) | HR 2.01 (1.68 to 2.4) | 20 more per 1000 (from 13 more to 27 more) | ⊕⊕⊕O MODERATE | CRITICAL |  |
| **MI (follow-up 8.2-11.03 years; assessed with: ICD-10)** | | | | | | | | | | | | |  |
| 2 | observational studies | serious^1^ | no serious inconsistency | no serious indirectness | no serious imprecision | none | 21164/999999  (2.1%) | 11487/999999  (1.1%) | HR 1.36 (1.18 to 1.56) | 4 more per 1000 (from 2 more to 6 more) | ⊕OOO VERY LOW | CRITICAL |  |
| **CV death (follow-up 5.66-14.75 years; assessed with: ICD-10 or ICD-9)** | | | | | | | | | | | | |  |
| 3 | observational studies | serious^1^ | serious^5^ | no serious indirectness | no serious imprecision | none | 45/683  (6.6%) | 33/3065  (1.1%) | HR 1.10 (0.82 to 1.47) | 1 more per 1000 (from 2 fewer to 5 more) | OOOO VERY LOW | CRITICAL |  |
| **all-cause mortality (follow-up 5.66-14.75 years; assessed with: ICD-10 or ICD-9)** | | | | | | | | | | | | |  |
| 4 | observational studies | serious^1^ | serious^6^ | no serious indirectness | no serious imprecision | none | 163/683  (23.9%) | 150/3065  (4.9%) | HR 1.08 (0.92 to 1.27) | 4 more per 1000 (from 4 fewer to 13 more) | OOOO VERY LOW | CRITICAL |  |

^1^ not analysis of publication bias; ^2^ linear positive association; ^3^ I2=82%; ^4^ HR=2.01; ^5^ I2=76%; ^6^ I2=87%.

Abbreviation: CI: Confidence interval; HR: Hazard ratio; CVD: cardiovascular disease; CAD: coronary artery disease; MI: myocardial infarction; TyG index: triglyceride and glucose index; CV mortality: cardiovascular mortality; ICD: International Classification of Diseases.**Figure S1**. Forest plot for the association between TyG index and risk of cardiovascular diseases (median vs. lowest). A. CAD; B. MI; C. CVD; D. All-cause mortality


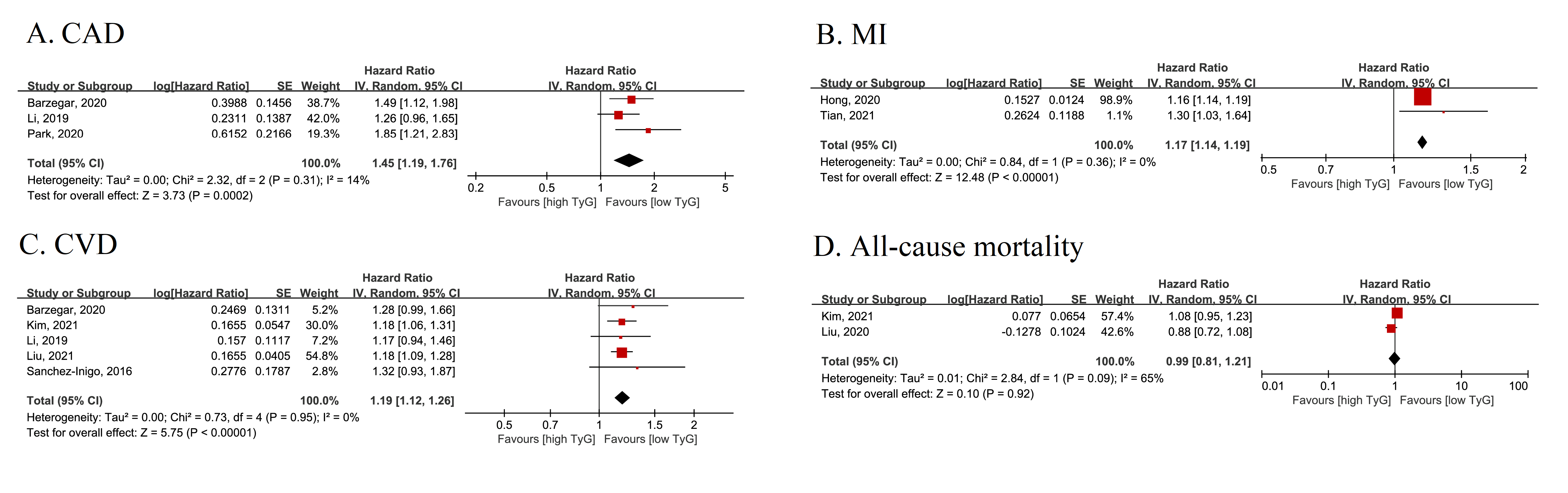


Abbreviation: TyG: Triglyceride-glucose; CAD: coronary artery disease; MI: myocardial infarction; CVD: cardiovascular disease.

**Figure S2**. Sensitivity analyses of association between TyG and cardiovascular diseases (categorical variables, highest vs. lowest) by omitting one study at once. A. CAD; B. CVD; C.CV death; D. All-cause mortality


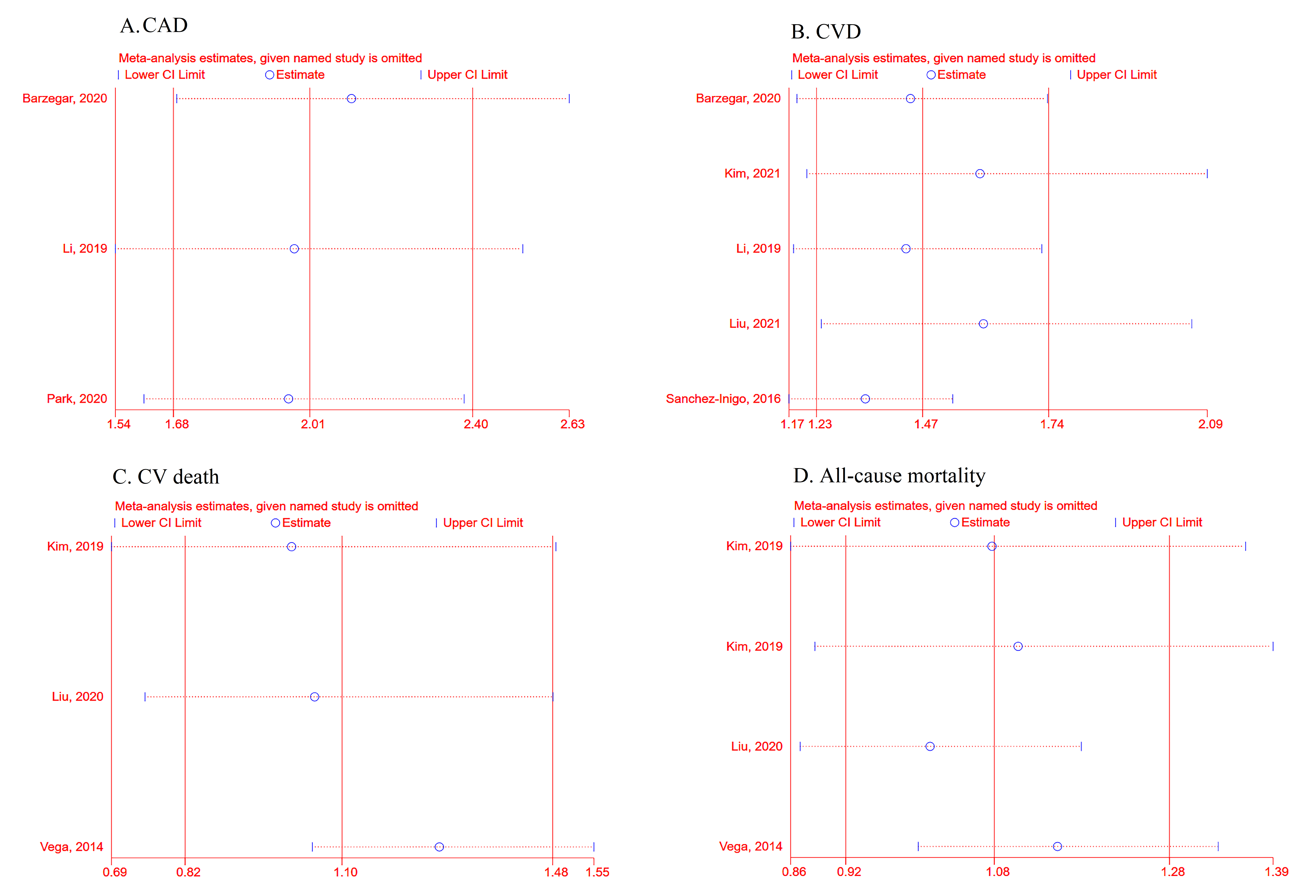


Abbreviation: TyG: Triglyceride-glucose; CAD: coronary artery disease; CVD: cardiovascular disease.

**Figure S3.** Forest plot for the association between TyG index and MI incidence in non-diabetes population, analyzed as category variables (highest vs. lowest).


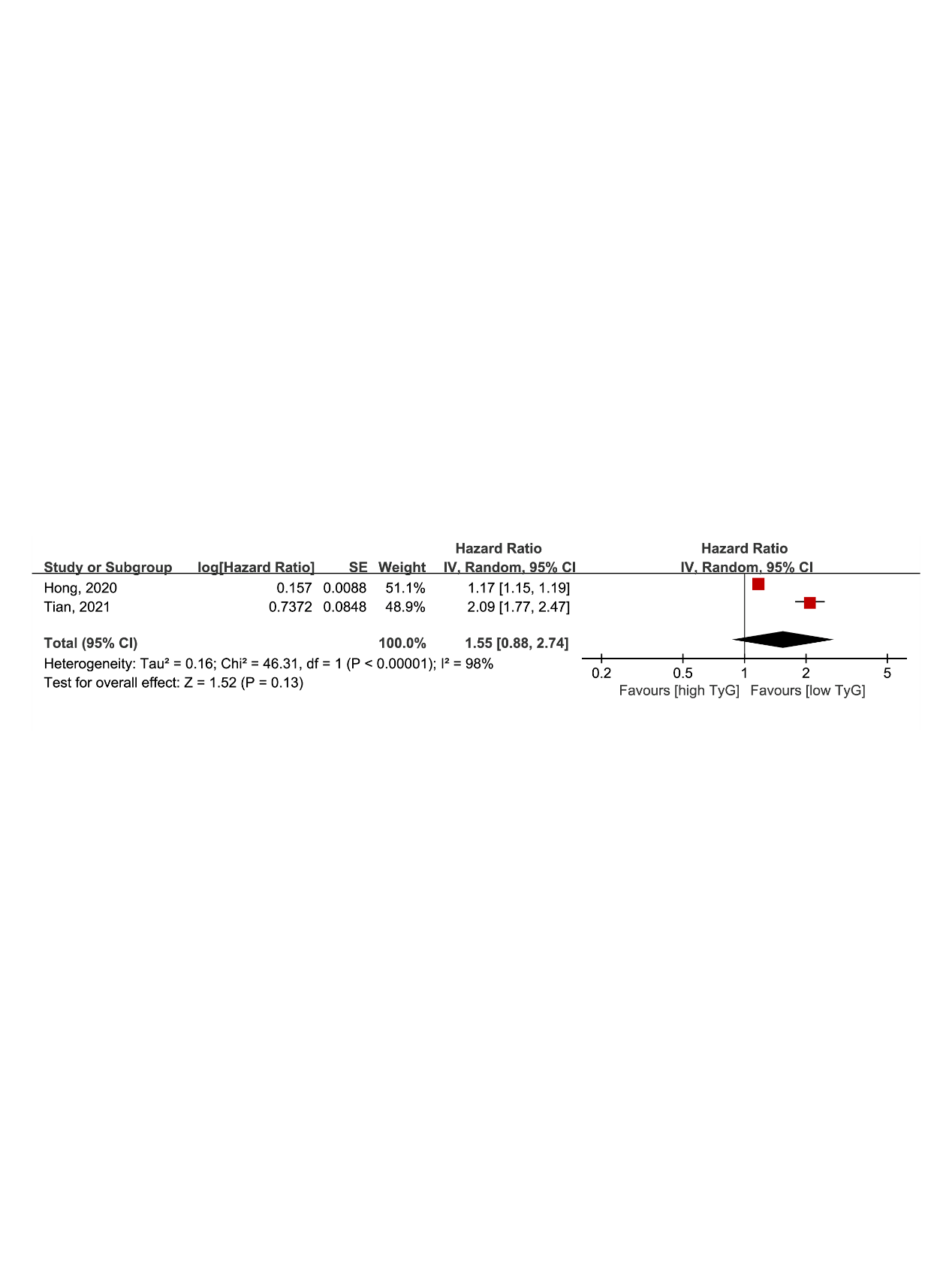


Abbreviation: TyG: Triglyceride-glucose; MI: myocardial infarction.
